# Establishing 3D organoid models from patient-derived conditionally reprogrammed cells to bridge preclinical and clinical insights in pancreatic cancer

**DOI:** 10.1186/s12943-025-02374-y

**Published:** 2025-06-03

**Authors:** Jin Su Kim, Chan Hee Park, Eunyoung Kim, Hee Seung Lee, Jinyoung Lee, Jeehoon Kim, Eun Hee Kam, Sanghee Nam, Moon Jae Chung, Jeong Youp Park, Seung Woo Park, Sangwoo Kim, Galam Leem, Seungmin Bang

**Affiliations:** 1 Division of Gastroenterology, Department of Internal Medicine, Severance Hospital, Yonsei University College of Medicine, 50-1 Yonsei-ro, Seodaemun-gu, Seoul, 03722 Republic of Korea; 2 Department of Internal Medicine, Graduate School of Yonsei University, Seoul, Republic of Korea; 3 Department of Biomedical Systems Informatics and Brain Korea 21 Project, Yonsei University College of Medicine, Seoul, Republic of Korea

**Keywords:** Pancreatic cancer, Conditionally reprogrammed cell (CRC) organoids, 3D organoid culture, Drug sensitivity screening, Precision medicine, Gemcitabine plus nab-paclitaxel (Abraxane), FOLFIRINOX

## Abstract

**Background:**

Pancreatic cancer is a highly lethal malignancy with limited treatment response. Despite advancements in treatment, systemic chemotherapy remains the primary therapeutic approach for over 80% of patients, with no established biomarkers to guide drug selection. Traditional two-dimensional (2D) culture models fail to replicate the tumor microenvironment, necessitating the development of more advanced models, such as three-dimensional (3D) organoid models.

**Methods:**

We established 3D organoid cultures using patient-derived conditionally reprogrammed cell (CRC) lines, originally cultured under 2D conditions. These CRC organoids were developed using a Matrigel-based platform without organoid-specific medium components to preserve the intrinsic molecular subtypes of the cells. Morphological, molecular, and drug sensitivity analyses were performed to compare the clinical responses of 3D CRC organoids with those of their 2D counterparts and clinical responses.

**Results:**

The 3D CRC organoids retained the molecular characteristics, transcriptomic and mutational profiles of the parental tumors and displayed distinct morphologies corresponding to cancer stages and differentiation. Drug response profiling of gemcitabine plus nab-paclitaxel (Abraxane) and FOLFIRINOX demonstrated that the 3D organoids more accurately mirrored patient clinical responses than the 2D cultures. Notably, the IC50 values for the 3D organoids were generally higher, reflecting the structural complexity and drug penetration barriers observed in vivo.

**Conclusion:**

Matrigel-based 3D organoid culture models provide a robust platform for pre-clinical drug evaluation, overcoming the limitations of 2D models. Although time- and resource-intensive, integrating both 2D and 3D platforms enables efficient initial screening and validation. This approach holds promise for identifying predictive biomarkers and advancing precision medicine in pancreatic cancer treatment.

**Supplementary Information:**

The online version contains supplementary material available at 10.1186/s12943-025-02374-y.

## Background

Pancreatic cancer is a highly lethal malignancy projected to become the second leading cause of cancer-related deaths by 2025 [1, 2]. Pancreatic ductal adenocarcinoma (PDAC) is characterized by a distinct set of recurrent genetic alterations that drive tumor initiation and progression. The most prevalent mutation occurs in the KRAS oncogene, detected in over 90% of PDAC cases and plays a central role in aberrant MAPK and PI3K signaling pathways, promoting cell proliferation and survival [3]. Additionally, loss-of-function mutations in key tumor suppressor genes: TP53, CDKN2A, and SMAD4 are frequently observed, occurring at rates of 60–70%, 30–50%, and 20–50%, respectively [3–5]. Despite notable advances in cancer treatment over the past decades, treatment outcomes for pancreatic cancer have not shown any significant improvement, with a 5-year survival rate of only 10% [1]. This stagnation is largely due to the limited efficacy of systemic chemotherapy, which is administered to over 80% of patients with pancreatic cancer. Current standard chemotherapy regimens, such as FOLFIRINOX (5-fluorouracil, folinic acid, irinotecan, and oxaliplatin) or gemcitabine plus nab-paclitaxel (Abraxane), do not extend patient survival up to 12 months [6–8]. As no biomarkers have been developed to predict the response to these regimens, physicians select the primary chemotherapy regimen based on general conditions of the patients or the physician’s preference. This contributes to the lack of improvement in patient response to chemotherapy. Therefore, advancing drug screening techniques using patient-derived samples is necessary.

Previously, conventional two-dimensional (2D) cell culture models were widely used to study pancreatic ductal adenocarcinoma (PDAC). These models are easy to handle, allow for continuous culture, and are cost-effective [9]. However, 2D cultures differ substantially from the original tumor in various aspects, including the tumor microenvironment, cell metabolism, and gene expression profiles [10–12]. To address these limitations, three-dimensional (3D) cell culture technologies have been developed to better replicate the biological features of the tumor environment in vivo. Several studies have reported differences in biological phenotypes, molecular mechanisms, and drug responses between 2D and 3D culture models [13, 14]. Organoid culture models, the most widely used 3D cell culture platform, closely replicate the morphology, gene and protein expression, cell polarity, and cellular metabolic heterogeneity of primary tumors. Importantly, they have demonstrated notable potential in advancing new drug development for precision and personalized medicine [15, 16].

Recently, we established a patient-derived conditional reprogramming cell line (CRC) that serves as a unique ex vivo model for personalized cancer therapy for PDAC [17, 18]. Since the cell line was initially established using 2D cell cultures, we developed an extracellular matrix component-based in vitro 3D cell printing culture model. This model more closely mimics the gene expression patterns of primary tumors compared to 2D cell cultures and reflects similar drug responses to those observed in clinical patients with PDAC [19].

In this study, we established a 3D organoid culture platform using patient-derived CRC lines that were previously grown in 2D, employing a Matrigel-based 3D organoid culture method. Notably, we avoided using organoid culture media components such as Wnt3a, R-Spondin-1, and Noggin, which are known to influence the molecular subtypes of cancer cells [20–25]. Then, we demonstrated the morphological, molecular characteristics of established CRC organoids and performed the drug sensitivity analyses to compare the clinical responses of 3D CRC organoids with those of their 2D counterparts and clinical responses.

## Methods

### Patients and tissue samples

This study was approved by the Institutional Review Board of Severance Hospital, Seoul, South Korea (No. 4-2019-0614). Written informed consent was obtained from all patients for sample collection and molecular analysis. The inclusion and exclusion criteria for patient selection are detailed in Supplementary Table S1. Pancreatic cancer tumor tissues were obtained through endoscopic ultrasound-guided fine-needle biopsy or surgical resection.

### Establishment of CRC organoids

In this study, pre-established patient-derived pancreatic cancer cell lines were used, employing a conditional reprogramming method [18]. Briefly, pancreatic cancer tumor tissues were obtained through endoscopic ultrasound-guided fine-needle biopsy or surgical resection, and fresh tumor tissues were cut into small pieces (2–4 mm) using dissection scissors. The tumor pieces were then subjected to enzymatic and mechanical digestion and resuspended to a single-cell level using a Human Tumor Dissociation Kit (Miltenyi Biotec, Germany) according to the manufacturer’s instructions. After digestion, the cell suspensions were filtered using a 40 µM-pore cell strainer (Corning, USA). Next, the cell suspensions were seeded on a feeder layer of lethally irradiated (30 Gy) J2 murine fibroblasts in F medium, consisting of 70% Ham’s F-12 nutrient mix (Hyclone, USA) and 25% complete Dulbecco’s Modified Eagle’s Medium, supplemented with 0.4 mg/mL hydrocortisone, 5 mg/mL insulin, 8.4 ng/mL cholera toxin, 10 ng/mL epidermal growth factor, 5% fetal bovine serum, 24 mg/mL adenine, 10 mg/mL gentamicin, and 250 ng/mL Amphotericin B. Additionally, the Rho-associated kinase inhibitor Y-27,632 (Sigma-Aldrich, USA) was added at a final concentration of 5 µM, and the cells were incubated at 37 °C in a humidified atmosphere with 5% CO_2_.

For CRC organoid culture, CRCs were mixed with 90% growth factor-reduced Matrigel (Corning, USA). For rapidly growing cells, the cell density was adjusted to 5,000 cells per 20 µL of 90% Matrigel, while for slower- growing cells, the cell density was set at 10,000 cells per 20 µL. The cells were thoroughly mixed with Matrigel, and 20 µL of the resulting mixture was aliquoted into each well of a 6-well cell culture plate, forming a total of seven dome structures. The cell suspension was allowed to solidify in the 6-well plates at 37 °C for 20 min. Subsequently, 4 mL of F medium was added to each well, and the medium was refreshed every 3–4 days. We harvested the organoids and proceeded with downstream assays or subculturing once more than 50% of the organoids in the culture exceeded 300 μm in size. We passaged the organoids multiple times to assess their stability over time.

### Paraffin embedding of CRC organoids

Approximately 2–4 weeks after seeding, depending on the cell type, the diameter of the CRC organoids reached 200–300 μm. The CRC organoids were transferred to a 15 mL tube, and 10 mL of chilled PBS was added. After gentle pipetting, the tube was incubated on ice for 5 min. The organoids were then centrifuged at 1500 RPM for 3 min, followed by careful removal of the supernatant. This procedure was repeated three times to efficiently isolate the CRC organoids from the Matrigel. Next, the CRC organoids were then fixed in 4% paraformaldehyde (PFA) at 4 °C overnight, dissociated from Matrigel, washed with PBS, and suspended in 3% ultra-low-gelling temperature (ULGT) agarose. Gelation occurred within 10–15 min at 4 °C. Each solidified 3% ULGT agarose CRC organoid button was transferred into the cap of a 5 mL snap tube. The cap, containing the 3% ULGT agarose CRC organoid button at the bottom, was then filled with 1% agarose containing 0.01% Erythrosine B, using the cap as a mold. The resulting agarose gel discs were removed from the caps and subjected to tissue processing for paraffin embedding [26–30].

### Immunofluorescence (IF) assay

For 2D CRC IF, CRC cells were seeded in 8-well culture slides at a density of 10,000 cells/well and incubated for 24 h. The cells were then fixed in 4% PFA for 15 min at room temperature and washed thrice with PBS. CRC organoid IF was performed on paraffin-embedded CRC organoid slides. After antigen retrieval by boiling the slides in 10 mM sodium citrate-buffered distilled water (pH 6.0) for 30 min in a 97 °C water bath, the slides were cooled under tap water for 30 min, followed by three washes with PBS. Both CRC 2D cells and organoids were blocked with 10% horse serum (Thermo Fisher Scientific, USA) diluted in PBS for 1 h and incubated with primary antibodies at room temperature for 1 h. The cells were then washed thrice with PBS and incubated with a secondary antibody for 30 min. The primary and secondary antibodies were diluted in 1% horse serum. After three additional washes with PBS, the slides were mounted using a mounting solution containing DAPI (Vector Laboratories, USA). IF images were acquired using an Olympus BF53 microscope (Olympus Life Sciences, Tokyo, Japan).

### KRAS, SMAD4, and TP53 mutation analysis

KRAS, SMAD4, and TP53 mutations in CRC 2D cells and organoids were analyzed using PCR. Genetic analysis of the KRAS gene was performed by PCR amplification of exon 1 (codons 12 and 13), while SMAD4 genetic analysis was conducted by PCR amplification of exons 2, 3, 6, and 12. DNA was extracted from CRC 2D cells and organoids using a QIAGEN QIAamp DNA Mini Kit (Hilden, Germany). For TP53, PCR amplification targeted exons 6, 7, 8, and 10, which encompass known mutation hotspots. All primer sets were specifically designed to verify the presence of mutations previously identified by next-generation sequencing (NGS) of the matched primary tumor tissue. PCR primers for KRAS sequencing were designed in-house as follows: forward (5′-AGGCCTGCTGAAAATGACTGA-3′) and reverse (5′-GGTCCTGCACCAGTAATATGCA-3′). PCR primers for SMAD4 sequencing were: exon 2 forward (5′-TTCCTTGCAACGTTAGCTGTTGTTT-3′) and exon 2 reverse (5′-TTGCATATTCTTCCAGAAATTCCCA-3′); exon 3 forward (5′-GTGTCTTGCATAATGTGACACATGAATAAAT-3′) and exon 3 reverse (5′-GAGATCCTTTTCCCTTTATGTTTCTTAGGAT-3′); exon 6 forward (5′-ACATCTATGAATGTACCATGTTAATGTCTTCTT-3′) and exon 6 reverse (5′-GCCCACATGGGTTAATTTGCTTT-3′); and exon 12 forward (5′-CTGATGTCTTCCAAACTCTTTTCTG-3′) and exon 12 reverse (5′-TGTATTTTGTAGTCCACCATC-3′). PCR primers for TP53 sequencing were: exon 6 forward (5′-CAGGCCTCTGATTCCTCACT-3′) and reverse (5′-CTTAACCCCTCCTCCCAGAG-3′); exon 7 forward (5′-CCACAGGTCTCCCCAAGG-3′) and reverse (5′-AAGTGGCTCCTGACCTGGAGTCTT-3′); exon 8 forward (5′-GCCTCTTGCTTCTCTTTTCC-3′) and reverse (5′-TAACTGCACCCTTGGTCTCC-3′); and exon 10 forward (5′-CAATTGTAACTTGAACCATC-3′) and reverse (5′-GGATGAGAATGGAATCCTAT-3′). After PCR amplification, the products were loaded onto a 2% agarose gel with a 6× loading dye (DYNEBIO, Republic of Korea) and analyzed by gel electrophoresis using a Gel Documentation System (Carestream, USA). Sanger sequencing was performed using the Cosmo GENETECH DNA sequencing service.

### Scanning electron microscopy (SEM)

CRC organoids were harvested and centrifuged at 1500 RPM at 4 °C for 3 min. The supernatant was discarded, and the CRC organoids were washed thrice with PBS for 10 min each. The CRC organoids were then fixed for 24 h in Karnovsky’s fixative (2% glutaraldehyde, 2% paraformaldehyde in 0.1 M phosphate buffer, pH 7.4) and washed twice for 30 min in 0.1 M PB. Post fixation was performed with 1% osmium tetroxide (OsO_4_) for 2 h, followed by dehydration in a gradually increasing ethanol series (from 50 to 100%) using a Critical Point Dryer (LEICA EM CPD300). Finally, the CRC organoids were coated with carbon using ion sputtering (LEICA EM ACE600) and observed under a field-emission scanning electron microscope (MERLIN, ZEISS).

### Targeted deep sequencing

The quality and quantity of purified DNA were assessed using fluorometry (Qubit, Invitrogen) and gel electrophoresis. Briefly, 500 ng of genomic DNA from each sample was fragmented by acoustic shearing using a Covaris S2 instrument, generating DNA fragments of 150–200 bp, which were then ligated to Illumina adapters and PCR-amplified. The samples were subsequently concentrated to 750 ng in 3.4 µl DW using a Speedvac machine (Thermo Scientific) and hybridized with RNA probes from the SureSelect XT Custom Panel Kit library (Supplementary Gene List) for 16–24 h at 65 °C. The targeted gene panel is presented in Supplementary Table S2. Following hybridization, the captured targets were isolated using biotinylated probe/target hybrids, streptavidin-coated magnetic beads (Dynabeads MyOne Streptavidine T1; Life Technologies Ltd.) and buffers. The selected regions were PCR-amplified using Illumina PCR primers. Library integrity was assessed using the Agilent TapeStation 4200 with High Sensitivity D1000 ScreenTape (Agilent, Santa Clara, CA) and quantified using the KAPA Library Quantification Kit (Kapa Biosystems). High-quality libraries were pooled and sequenced on the Illumina NovaSeq6000 platform (Illumina) using 150 bp paired-end sequencing following the manufacturer’s protocols. Image analyses were performed using NovaSeq6000 control software (v1.3.1), and the output base calling data was de-multiplexed with bcl2fastq (v2.20.0.422) generating FastQC files.

To systematically assess genetic concordance between samples, genotype concordance analysis was performed using BAMixChecker, including all matched patient samples: tumor, CRC, and organoid. Default parameters were applied, and pairs with concordance scores greater than 0.7 were considered genetically matched.

Somatic variant calling was conducted using GATK Mutect2 (v4.1.2.0) for primary tumors, CRCs and organoid samples from each patient. Candidate variants from a panel of 83 targeted genes were annotated using SnpEff (v4.3) to characterize their genomic features. Somatic mutations were further annotated based on entries from the Catalogue of Somatic Mutations in Cancer (COSMIC). Additionally, the potential effects of these mutations on biological function were predicted using PROVEAN (v1.1.5) and SIFT (v6.2.1). To ensure the reliability of somatic variants, strict filtering criteria recommended by Mutect2 were applied. Variants were retained only if they met all of the following conditions: at least 5 reads supporting the alternative allele, a total read depth of at least 20 at the site, and an allele frequency (AF) of 0.05 or higher. These filters minimized sequencing errors and low coverage variants, ensuring the analysis focused on high-confidence mutations. By maintaining clear and consistent thresholds, the accuracy and credibility of downstream mutation analyses were improved. Concordance mutations are defined as somatic mutations detected in primary tumors, CRCs, and organoid samples derived from the same patient. Organoid-specific mutations are detected only in organoids, but absent in paired CRCs or primary tumors. Similarly, mutations uniquely identified as high-confidence somatic variants identified in CRC or primary tumor samples, but absent in the organoid are defined as tumor/CRC-specific mutations.

### RNA sequencing

The libraries were prepared for 151 bp paired-end sequencing using TruSeq Stranded mRNA Sample Preparation Kit (Illumina, CA, USA). The mRNA molecules were purified and fragmented from 1 µg of total RNA using oligo (dT) magnetic beads. The fragmented mRNAs were synthesized as single-stranded cDNAs through random hexamer priming, which was subsequently used as a template for second strand synthesis to generate double-stranded cDNA. The cDNA libraries underwent a sequential process of end repair, A-tailing and adapter ligation, followed by PCR (Polymerase Chain Reaction) amplification. Library quality was evaluated using the Agilent 2100 BioAnalyzer (Agilent, CA, USA), and quantified using the KAPA Library Quantification Kit (Kapa Biosystems, MA, USA) per the manufacturer’s protocol. Following cluster amplification of denatured templates, sequencing was performed in a paired-end (2 × 151 bp) format using Illumina NovaSeq6000 (Illumina, CA, USA). The adapter sequences and low quality regions (Phred quality score < 20) were trimmed, and reads shorter than 50 bp were removed using cutadapt v.2.8 [31]. After adapter trimming and removal of low-quality sequences with Trimmomatic, and elimination of mouse contamination from CRC samples with Disambiguate, cleaned reads were aligned to the human genome using STAR. Gene expression levels were quantified using cufflinks, yielding values such as FPKM. To examine gene expression similarities and differences across samples, principal component analysis (PCA) was performed using the prcomp function in R.

### Cell viability and drug sensitivity assessment in 2D and 3D cultures

Cell viability and drug sensitivity were assessed under 2D and 3D organoid culture conditions to determine the drug response of CRC lines. In the 2D culture condition, CRC cells were seeded at a density of 5,000 per well in 48-well plates. Cells were treated 24 h post-seeding with various concentrations of a gemcitabine plus nab-paclitaxel (Abraxane) combination (1X Dose: gemcitabine, 1 µM; nab-paclitaxel [Abraxane], 0.125 µM) and FOLFIRINOX (1X Dose: 5-FU, 34.3 µM; irinotecan, 0.4 µM; oxaliplatin 0.32 µM) for 72 h. Drugs were administered at a fixed ratio, reflecting the clinically relevant dose used in patients. The drug ratios for FOLFIRINOX were determined based on a previous study that applied FOLFIRINOX to pancreatic cancer organoids following the same rationale [32]. Cell viability was assessed using the CellTiter-Glo 2D reagent (Promega, USA) according to the manufacturer’s instructions. For 3D organoid culture, 5,000 CRC organoids were premixed with 90% Matrigel and seeded in 48-well cell culture plates. After 4 days of culture for organoid structural stabilization, CRC organoids were treated with the same drug combinations used in the 2D culture for 72 h. At the time of drug treatment, organoid diameter averaged 88.07 ± 26.31 μm. Cell viability was assessed using the CellTiter-Glo 3D reagent (Promega, USA) according to the manufacturer’s instructions. Inhibitory concentration (IC50) values were calculated using CompuSyn software (Version 1.0, USA). All assays were performed in triplicate.

### Clinical response to chemotherapy

Clinical response to chemotherapy was assessed every 2 to 3 months following treatment initiation using imaging modalities such as computed tomography (CT) or magnetic resonance imaging (MRI). Assessments were conducted in accordance with the Response Evaluation Criteria in Solid Tumors (RECIST) version 1.1, a standardized method for evaluating treatment response in solid tumors. According to RECIST version 1.1, partial response (PR) was defined as at least a 30% decrease in the sum of diameters of target lesions, relative to baseline measurements. Progressive disease (PD) was defined as at least a 20% increase in the sum of diameters of target lesions, taking the smallest recorded sum as the reference (including baseline if applicable). Stable disease (SD) was defined as neither sufficient shrinkage to qualify for PR nor sufficient increase to qualify for PD. The tumor size change percentage was calculated at the time of the first response evaluation by comparing changes in the sum diameter of target lesions to baseline measurements. The best response was defined as the most favorable tumor response observed at any time during the treatment period. Tumor size change in this analysis was determined by measuring the difference between tumor size at the first response evaluation and the baseline measurement.

### Resources and agents

All antibodies and reagents used in this study are presented in Supplementary Table S3.

### Statistical analysis

All statistical analyses were performed using Microsoft Excel and SigmaPlot software (Version 15.0, USA). IC50 values were calculated using CompuSyn software (Version 1.0, USA). To compare IC50 values between independent groups, two-tailed unpaired t-tests with Welch’s correction (to account for unequal variances) were applied. Linear regression analysis was performed to evaluate correlations between IC50 values and tumor size changes and between COSMIC gene expressions of tumor, CRCs and organoids. Additionally, Fisher’s exact test was used to assess associations between organoid morphological types, cancer stages, and tumor differentiation. Statistical significance was defined as *p* < 0.05.

## Results

### Establishment of matrigel-based CRC organoids

**Fig. 1:**
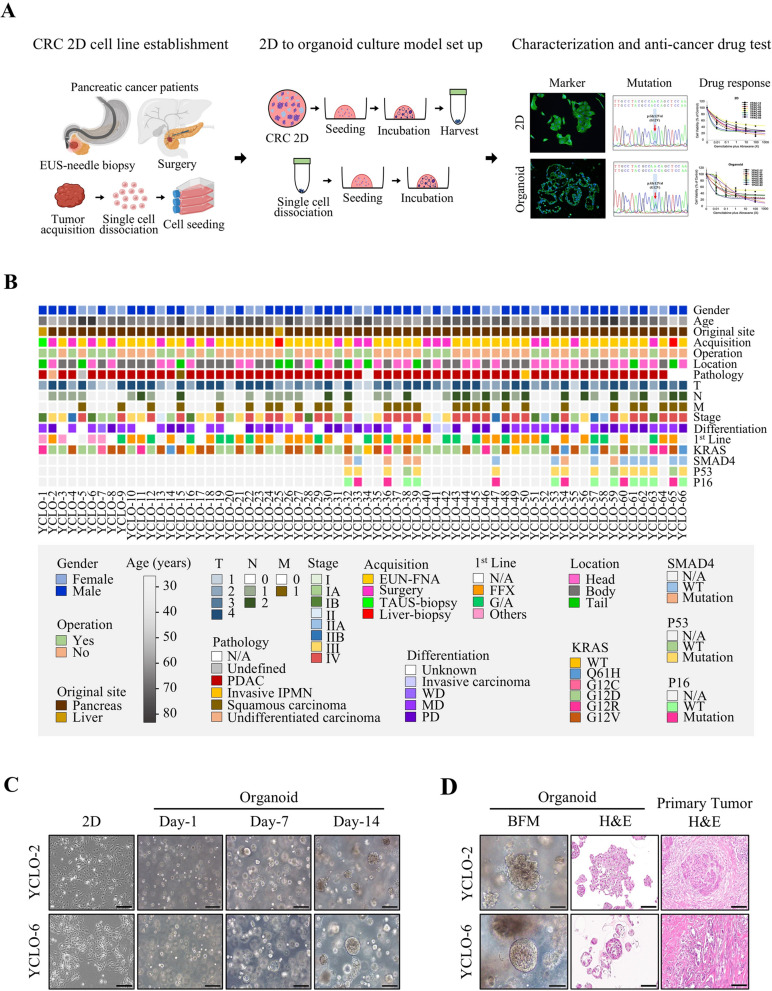
The establishment of a Matrigel-based 3D organoid culture model for pancreatic cancer utilized established CRC cell lines derived from patients with pancreatic cancer. (**A**) A research scheme for transitioning from 2D to organoid culture models was developed in our laboratory, successfully producing CRC organoids. These organoids were characterized and utilized by assessing their morphological phenotypes, marker expression, target gene mutations, and multi-drug screening. (**B**) Clinical information heatmaps for established CRC organoids. (**C**–**D**) Established CRC organoids showing morphological and histological correspondence with primary tumors. Scale bar: 200 μm. (**C**) CRC cell line 2D images and growing CRC organoids. (**D**) Representative matching images of CRC organoids under bright-field microscopy (BFM) and hematoxylin and eosin (H&E) staining, along with H&E staining images of primary tumor tissues. Scale bar: 100 μm. Abbreviations: CRC, conditionally reprogrammed cell lines; BFM, bright-field microscopy; H&E, hematoxylin and eosin staining; N/A, not available

CRC cells were established using a previously described protocol [17, 18]. We successfully developed 66 CRC organoids from 86 previously generated CRC cells. Additionally, we validated the molecular features and mutation profiles of these organoids in comparison to the original CRCs and evaluated their drug sensitivities to FOLFIRINOX and gemcitabine plus Abraxane under both culture conditions (Fig. 1A). The clinical information and baseline characteristics are shown in Fig. 1B.

Under 2D culture conditions, CRC cells displayed a monolayer growth pattern with dispersed cell morphology. Conversely, in 3D organoid cultures, CRC cells began proliferating by Day 1 and formed spherical structures by Day 7. By Day 14, most CRC cells had developed a 3D organoid structure (Fig. 1C and Supplementary Fig. S1). Most organoid cultures were terminated within 2–4 weeks once the organoids reached a size of 200–300 μm. If organoid shapes did not form within 2 weeks, they were discarded. The established CRC organoids exhibited dense cellular clusters, closely resembling the morphological and histological structures of primary tumor tissues (Fig. 1D and Supplementary Fig. S2). Unlike the dispersed arrangement observed in 2D cultures, the 3D organoids formed multicellular structures that replicated original tumor histology more effectively.

### Molecular characterization of CRC organoids

**Fig. 2:**
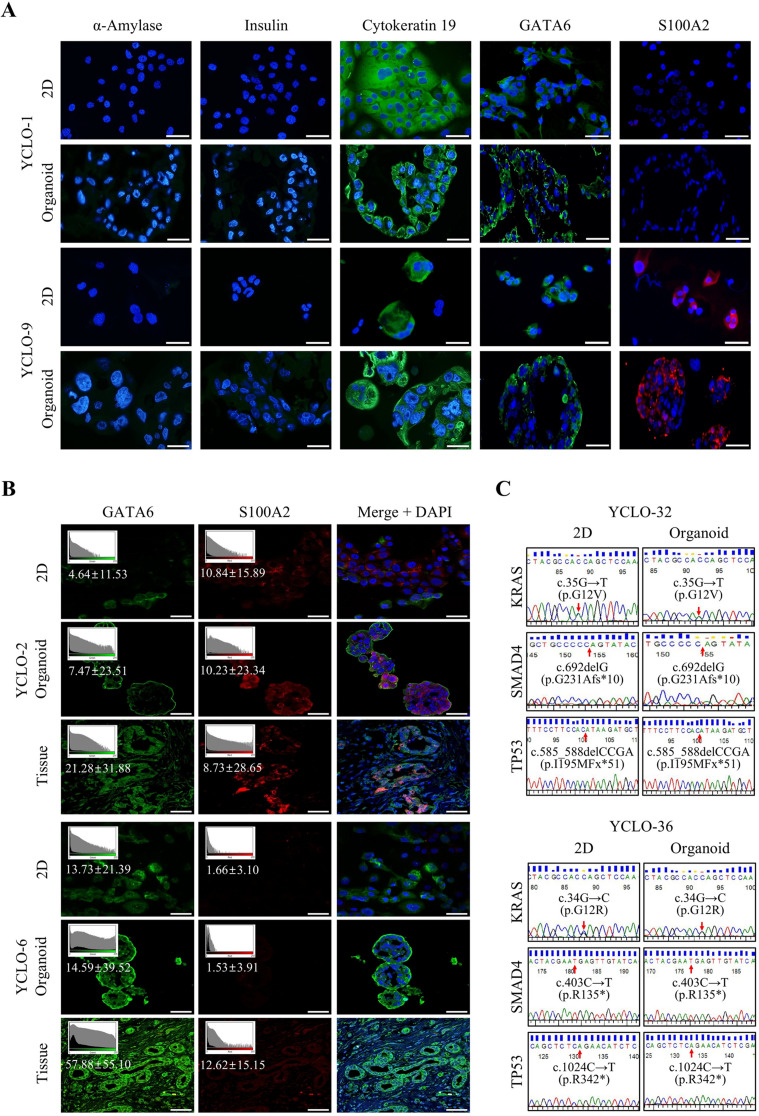
Characterization of established CRC organoids. (**A**) Immunofluorescence (IF) staining of representative 2D and organoid models using selected marker antibodies. IF staining was performed for cytokeratin 19 (an epithelial marker), α-amylase and insulin (markers for normal pancreas cells), and GATA6 (classical subtype marker), and S100A2 (basal subtype marker). Scale bar: 50 μm. (**B**) Validation of subtype marker expression patterns among parental tumor tissues, 2D CRCs, and corresponding organoids. Immunofluorescence staining for the classical marker GATA6 and basal marker S100A2 was performed on CRC 2D cultures, organoids, and matched primary tumor tissues from YCLO-2 and YCLO-6. Fluorescence intensity (mean ± SD) showed consistent expression patterns between 2D and organoid cultures, closely matching the original tissues and supporting subtype-specific feature preservation. Scale bar: 50 μm. (**C**) Representative sanger sequencing results showing identical mutations in KRAS (c.35G→T, c.34G→C), SMAD4 (c.692delG, c.403 C→T), and TP53 (c.585_588delCCGA, c.1024 C→T) in matched 2D and organoids. Red arrows indicate mutation sites. Abbreviations: YCLO, YPAC cell line organoid; Mut, mutation; Del, deletion

We confirmed that CRC organoids preserved the molecular characteristics of the original CRCs by comparing marker protein expression. The expression patterns of cytokeratin 19 (an epithelial cell marker), GATA6 (a classical subtype marker), and S100A2 (a basal-like subtype marker) were consistent between 2D and 3D CRC organoids (Fig. 2A and Supplementary Fig. S3). This finding indicated that the molecular phenotype of CRCs remained unchanged during the 3D conversion. We also validated the consistency of molecular subtypes among parental tumor tissues, 2D CRCs, and their corresponding organoids (Fig. 2B). Additionally, the absence of insulin, an islet cell marker, and α-amylase, an acinar cell marker, confirmed the purity and specificity of CRCs in both models.

We assessed the mutational profiles of CRC organoids by performing targeted sequencing of key pancreatic cancer-related genes, including KRAS, SMAD4, and TP53. Both 2D and 3D CRC models demonstrated consistent mutational profiles of these genes (Fig. 2C and Supplementary Fig. S4). Based on prior evidence that 2D CRCs accurately reflect primary cancer mutational profiles [17, 18], we concluded that CRC organoids faithfully maintain the genetic characteristics of primary cancers. To further substantiate this, we compared KRAS, SMAD4, and TP53 mutations across tumor tissues, 2D CRCs, and the corresponding CRC organoids from 15 patients. Our analysis confirmed that the identical mutations were consistently detected at the same loci in all matched samples (Supplementary Table S4). These findings highlight that the Matrigel-based 3D organoid culture system effectively preserves both the phenotypic and genotypic features of CRCs.

### Mutation profiling of CRC organoids

**Fig. 3:**
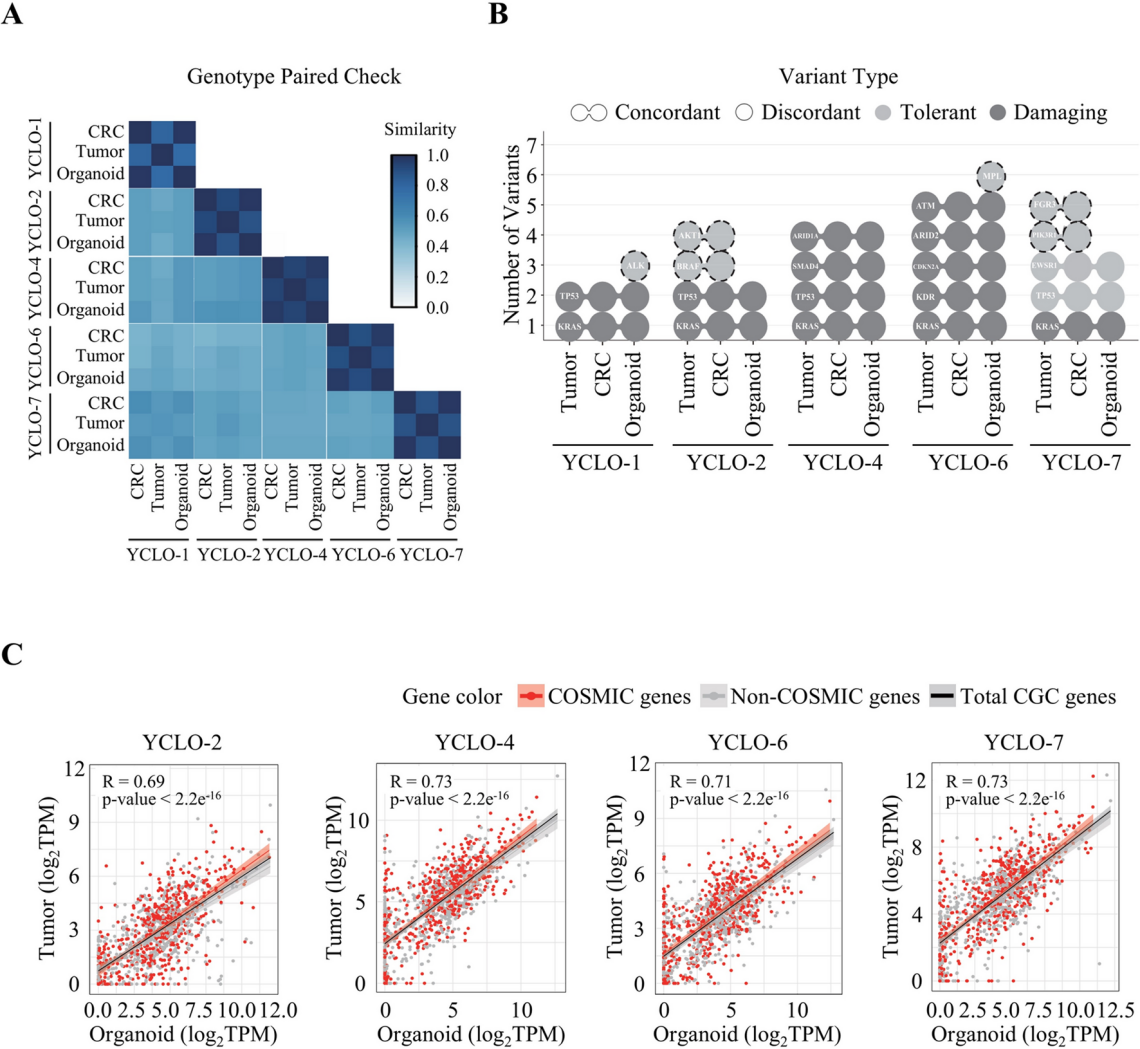
Pairwise genetic similarity of sample types. (**A**) Heatmap representing the concordance ratio of germline single-nucleotide polymorphisms (SNP) among three sample types—CRC, tumor, and organoid—across five individual samples. Similarity scores are calculated based on pairwise SNPs concordance, with values ranging from 0 to 1; higher scores (closer to 1) indicate greater genetic similarity and are shown in blue, while lower scores are shown in white. All five samples contain the three sample types, allowing assessment of genetic concordance and validation of patient-derived models relative to their original tissues. The results demonstrate strong genetic relationships between tumor, CRC, and organoid samples, confirming the genetic fidelity of patient-derived models. (**B**) Somatic mutation concordance across matched primary tumor, CRC, and organoid. Each circle represents a somatic mutation, with connected circles indicating mutations shared between two or more sample types, and single circles representing mutations unique to one sample type. Mutations are color-coded by predicted functional impact: light grey denotes tolerated mutations (e.g., synonymous or intronic), and dark grey indicates deleterious mutations (e.g., missense or frameshift). A dashed line highlights this exception. Organoid-specific and Tumor/CRC-specific mutations were also identified within the three patient-matched triplets. (**C**) Correlation between tumor and organoid gene expression profiles across four patient-derived samples. Each scatter plot shows the log₂-transformed TPM (Transcripts Per Million) values of CGC genes in matched tumor and organoid samples. Red dots represent genes listed in the COSMIC database, while grey dots represent non-COSMIC genes among the CGC gene set. The black regression line corresponds to the linear fit across all CGC genes, with a shaded area indicating the 95% confidence interval. Pearson correlation coefficients (R) and associated p-values are reported in each panel. COSMIC genes demonstrate strong concordance between tumor and organoid expression, indicating a strong concordance between tumor and organoid expression profiles for cancer-relevant genes

To more precisely assess whether the mutation profiles of established CRC organoids align with those of their corresponding parental tumor tissues, we performed targeted deep sequencing of 348 key oncogenes on five matched pairs of tumor tissues, CRCs, and CRC organoids (Supplementary Table S2). To confirm the origin and genetic fidelity of patient-derived models, we analyzed genetic variants across primary tumors, CRCs, and corresponding organoids. Germline single-nucleotide polymorphism (SNP) similarity was assessed to verify the matched origin of each model with its parental tumor tissue. Clustering analysis based on germline SNP similarity revealed that samples derived from the same patient, clustered together, demonstrating high genetic concordance across parental tumor tissues, CRCs, and corresponding organoids (Fig. 3A).

Somatic mutations were also identified. Initial mutation calling detected 22 somatic mutations per set, with 16 mutations shared among primary tumor tissues, CRCs and CRC organoids, leaving four tumor/CRC-specific mutations and two organoid-specific mutations (Fig. 3B). Shared mutations included key oncogenic drivers such as KRAS (5/5 samples), TP53 (4/5 samples), and SMAD4 (1/5 samples). Tumor- and CRC-specific mutations were mostly synonymous or low-impact variants in genes including AKT1 (YCLO-2), BRAF (YCLO-2), FGFR3 (YCLO-7), and PIK3R1 (YCLO-7). More detailed allele frequency analysis revealed that organoid-specific mutations in ALK (YCLO-1) and MPL (YCLO-6) were missense mutations predicted to be tolerated, indicating low functional impact. Notably, MPL (YCLO-6) exhibited a low variant allele frequency (VAF, 0.053) even in organoid cultures, suggesting that this mutation likely has limited biological significance. These findings demonstrate the high genetic similarity within each patient’s sample set, reinforcing the reliability of CRCs and CRC organoids as representative models of primary tumors.

### Transcriptomic profiling of CRC organoids

To further validate the molecular similarities among tumor tissues and their corresponding CRCs and CRC organoids, we performed RNA sequencing was conducted on four matched pairs of tumor tissues, CRCs, and CRC organoids. Principal component analysis (PCA) was performed to assess global transcriptomic similarities and differences across samples. The resulting PCA plot demonstrated that each CRC and CRC organoid clustered closely with their corresponding tumor tissue, indicating that CRC organoid models effectively recapitulate the transcriptomic profiles of their parental tissues (Supplementary Fig. S5). To further examine concordance in cancer-related gene expression, pairwise correlation analyses were conducted using gene sets curated from the Catalogue of Somatic Mutations in Cancer (COSMIC) and the Cancer Gene Census (CGC). Strong correlations were observed across all pairwise comparisons (tumor-CRC, tumor-organoid, and CRC-organoid), with Pearson correlation coefficients ranging from 0.69 to 0.89 (Fig. 3C and Supplementary Fig. S6). These findings provide additional validation that key cancer gene expression patterns in tumor tissues are well preserved in both CRCs and CRC organoids.

### Distinct morphological types of CRC organoids

**Fig. 4:**
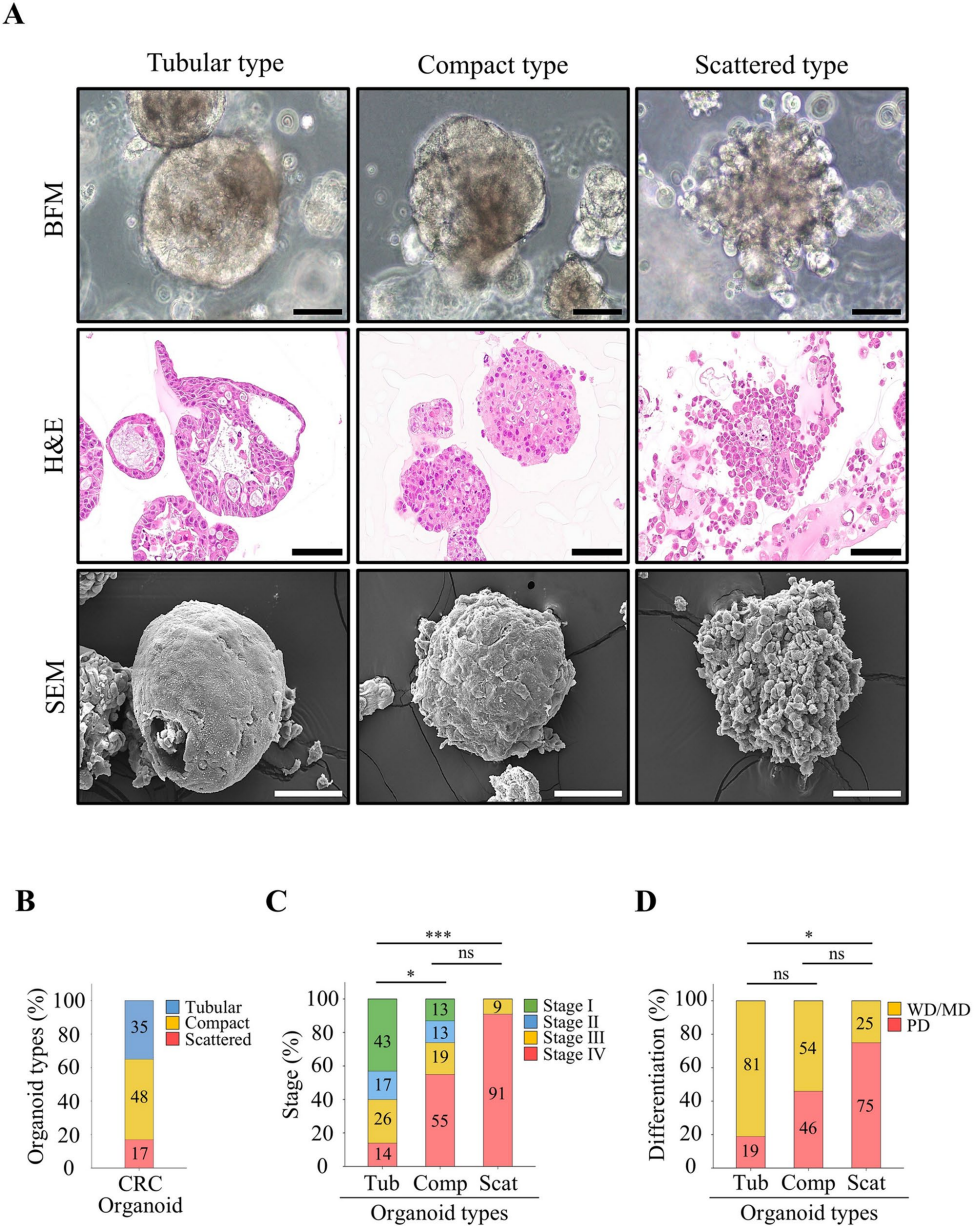
Various morphological types of established CRC organoids. (**A**) CRC organoids displayed compact, tubular, and scattered morphologies. Scale bars: 100 μm. (**B**) Distribution of organoid morphologies among the established CRC organoids. Compact-type organoids were the most common (48%), followed by tubular (35%) and scattered (17%) types. (**C**) A comparison of organoid types with cancer stages revealed an increase in scattered types as cancer stages progressed, while tubular types decreased. (**D**) A comparison of organoid types with cancer differentiation showed that poorly differentiated (PD) tumors were associated with increased prevalence of scattered types, followed by compact and tubular types. Fisher’s exact test was performed to evaluate associations between organoid morphological types, cancer stages, and tumor differentiation. Abbreviations: BFM, bright-field microscopy; H&E, hematoxylin and eosin staining; SEM, scanning electron microscopy; WD, well-differentiated; MD, moderately differentiated; PD, poorly differentiated; ns, not significant. *P < 0.05. ***P < 0.001

Previous studies reported that normal pancreatic organoids exhibit cystic structures, while tumor organoids display cystic and compact structures [33, 34]. We observed distinct morphological types in CRC organoids. Using bright-field microscopy, cross-sectional imaging, and SEM, we classified CRC organoids into three types: tubular, compact, and scattered (Fig. 4A and Supplementary Fig. S7). Tubular organoids exhibited elongated hollow structures with epithelial-lined lumens. Compact organoids displayed dense, spherical structures with smooth, rounded surfaces, while scattered organoids formed loosely packed, irregular structures. Compact organoids were the most prevalent (48%), while scattered organoids were the least common (17%) (Fig. 4B). Intriguingly, scattered organoids were more prevalent in advanced cancer stages and poorly differentiated cancers compared to tubular and compact types (Fig. 4C-D). However, no significant differences in sex, age, tumor location, or KRAS mutation type were observed among the morphological subtypes (Supplementary Fig. S8).

### Drug screening in 2D CRCs and 3D CRC organoids aligned with clinical response

**Fig. 5:**
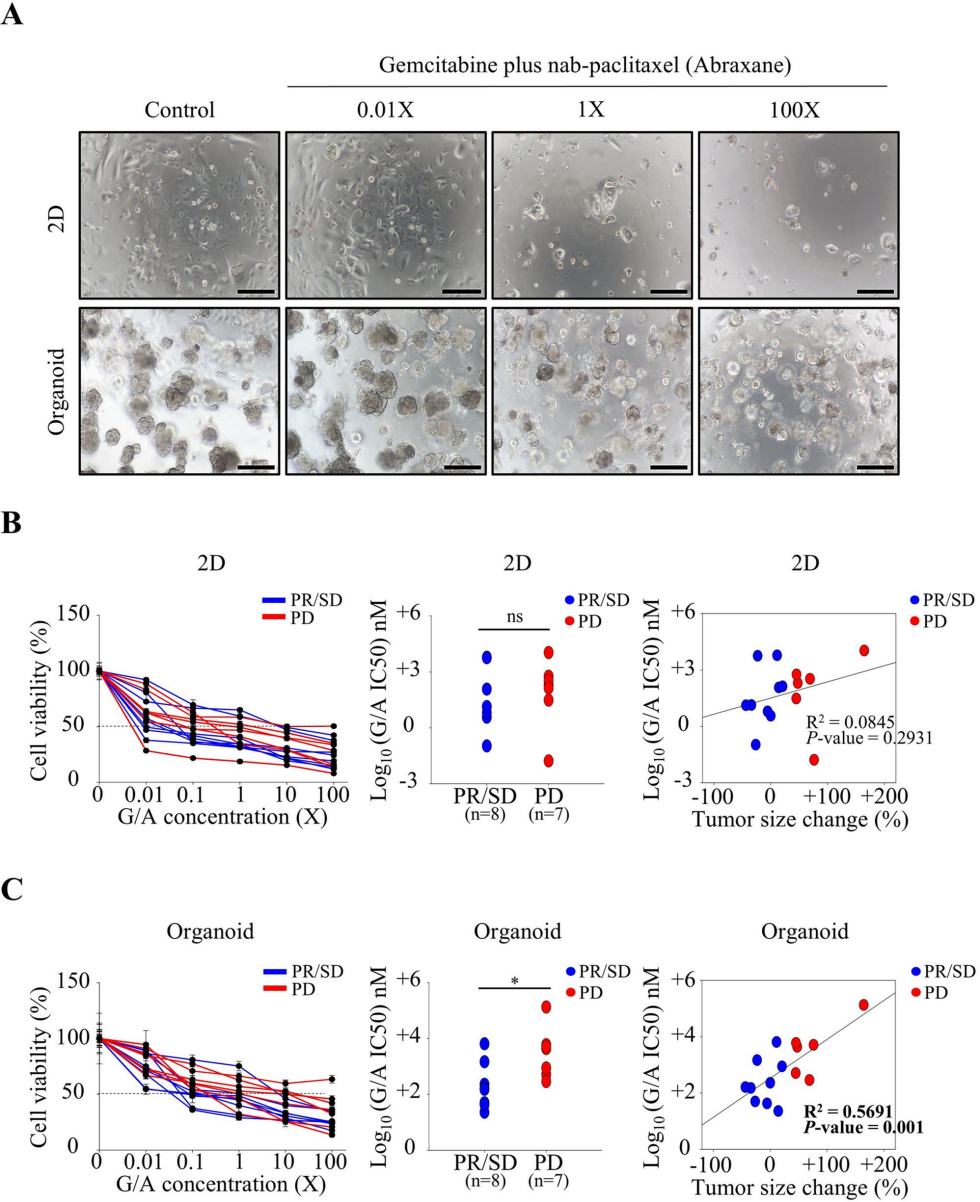
G/A combination drug response of established CRC cell lines in 2D and organoid culture conditions. (**A**) Representative bright-field microscopy (BFM) images of CRC cell lines treated with increasing concentrations (0.01X, 1X, 100X) of G/A under 2D and organoid culture conditions. Scale bar: 200 μm. (**B**) In the 2D culture condition, cell viability curves (left), IC50 values (middle), and their correlation with tumor size change (right) were analyzed between the PR/SD and PD groups. No statistically significant differences or correlations were observed. (**C**) In the organoid culture condition, IC50 values were significantly lower in the PR/SD group compared to the PD group (*P* < 0.05), and a significant positive correlation was observed between IC50 values and tumor size change (R² = 0.5691, *P* = 0.001), supporting the clinical relevance of the organoid model. Two-tailed unpaired t-tests with Welch’s correction (accounting for unequal variances) were conducted to compare IC50 values between independent groups. Linear regression analysis was performed to examine the correlation between IC50 values and tumor size changes. Abbreviations: G/A, gemcitabine plus nab-paclitaxel (Abraxane); IC50, inhibitory concentration 50; PR, partial response; SD, stable disease; PD, progressive disease; ns, not significant. *P < 0.05

**Fig. 6:**
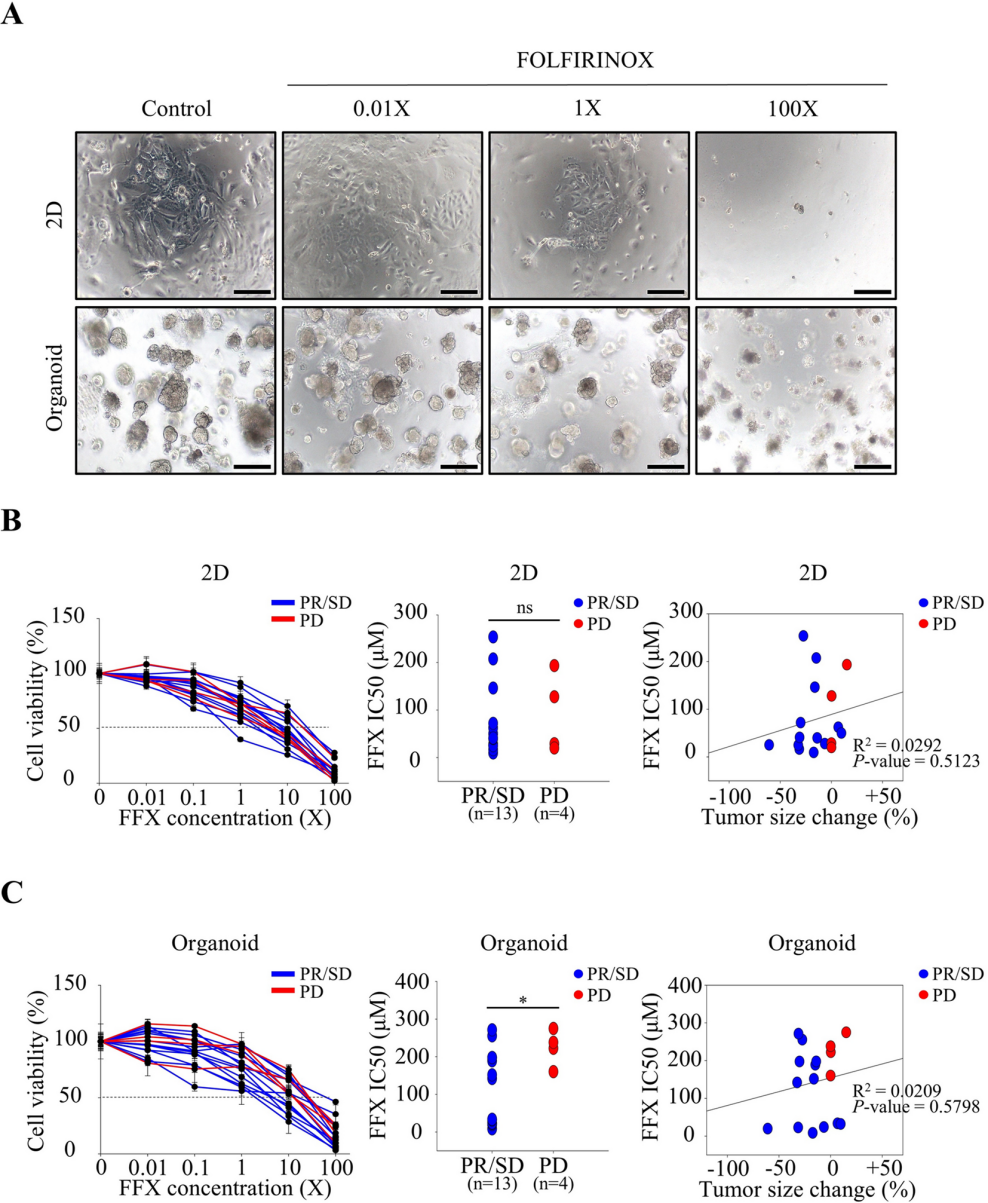
FOLFIRINOX combination drug response of established CRC cell lines in 2D and organoid culture conditions. (**A**) Representative bright-field microscopy (BFM) images showing the morphological responses of patient-derived CRC cell lines to increasing concentrations (0.01X, 1X, 100X) of FOLFIRINOX under 2D and organoid culture conditions. Scale bar: 200 μm. (**B**) In the 2D culture condition, cell viability curves (left), IC50 values (middle), and correlation with tumor size change (right) showed no significant differences between the PR/SD and PD groups. (**C**) In the organoid culture condition, IC50 values were significantly lower in the PR/SD group compared to the PD group (*P* < 0.05), while correlation analysis between IC50 values and tumor size change was not statistically significant. Two-tailed unpaired t-tests with Welch’s correction (accounting for unequal variances) were conducted to compare IC50 values between independent groups. Linear regression analysis was performed to examine the correlation between IC50 values and tumor size changes. Abbreviations: FFX, FOLFIRINOX; IC50, inhibitory concentration 50; PR, partial response; SD, stable disease; PD, progressive disease; ns, not significant. *P < 0.05

Finally, to evaluate the utility of drug screening in 2D and 3D CRC models, patient-derived 2D CRCs and 3D CRC organoids were treated with the same chemotherapeutic agents, gemcitabine plus nab-paclitaxel (Abraxane) (G/A) or FOLFIRINOX, used as first-line therapy. The responses of these models were then assessed to determine their consistency with the clinical responses observed in patients. Among the 66 patients from whom both 2D CRCs and 3D CRC organoids were established, 15 received G/A as first-line therapy, while 17 received FOLFIRINOX. These patient-derived 2D CRCs and 3D CRC organoids were treated with the same regimen in a ratio similar to that administered to the patients (Figs. 5A and 6A). In the 2D culture model, responses to G/A and FOLFIRINOX were inconsistent and did not correlate with the patients’ clinical outcomes (Figs. 5B and 6B). Interestingly, when the same CRCs were treated in 3D organoid formation, the drug responses closely aligned with the clinical results (Figs. 5C and 6C). Unlike the G/A treatment group, no statistically significant correlation between IC50 values and tumor size changes was observed in the FFX treatment group. This discrepancy may be attributed to the lower number of PD cases with measurable tumor growth in the FFX group (2/17) compared to the G/A group (7/15), as half of the PD cases in the FFX group were due to the appearance of new lesions rather than an increase in tumor size. Nonetheless, the statistically significant difference in IC50 values between response groups, even within the FFX treatment group, supports the conclusion that the 3D CRC organoid model demonstrated drug response patterns that accurately reflected patient sensitivity or resistance to G/A and FOLFIRINOX. These results confirmed that the Matrigel-based 3D organoid culture model provides a more reliable prediction of patient drug responses compared to the 2D culture-based drug test.

Moreover, the IC50 values for G/A and FOLFIRINOX were generally higher in 3D CRC organoids than in 2D CRCs (Tables 1 and 2), likely due to differences in drug exposure among cancer cells within the structural complexity of 3D organoids. In a few samples, however, higher IC50 values were observed in 2D cultures, which might be explained by hypoxia or limited nutrient supplement within the inner regions of 3D organoids, as these factors could have affected overall cell viability and drug response. In contrast, cancer cells in single-layer 2D cultures were uniformly exposed to the drugs. This finding suggests that 3D organoid models better mimic the tumor microenvironment in patients, offering a closer representation of in vivo conditions.

**Table 1:** Overview of the first G/A response in established CRC cell line 2D and organoid culture conditions

No	Samples	Best Response	Total Chemotherapy Cycles	Chemotherapy Cycles until Best Response	Tumor Size Change	2D IC50 (nM)	Organoid IC50 (nM)
1	YCLO-9	PD	2 Cycle	2 Cycle	+ 164.4%	11010.17	134043.74
2	YCLO-12	SD	6 Cycle	2 Cycle	0.0%	3.70	229.75
3	YCLO-16	SD	14 Cycle	2 Cycle	+ 11.1%	6144.15	6476.85
4	YCLO-19	PR	4 Cycle	2 Cycle	− 43.6%	13.50	162.05
5	YCLO-20	SD	9 Cycle	2 Cycle	− 23.1%	5785.55	1473.13
6	YCLO-22	PD	2 Cycle	2 Cycle	+ 45.2%	589.00	5977.93
7	YCLO-29	PD	2 Cycle	2 Cycle	+ 47.4%	205.35	4364.70
8	YCLO-36	PR	21 Cycle	2 Cycle	− 33.3%	0.11	50.09
9	YCLO-42	PD	2 Cycle	2 Cycle	+ 44.8%	30.69	511.21
10	YCLO-43	SD	4 Cycle	2 Cycle	+ 14.1%	119.28	22.91
11	YCLO-45	SD	4 Cycle	2 Cycle	− 5.6%	6.34	42.71
12	YCLO-48	PD	2 Cycle	2 Cycle	+ 76.2%	0.02	5127.16
13	YCLO-52	PD	3 Cycle	2 Cycle	+ 68.9%	343.13	288.36
14	YCLO-57	PR	8 Cycle	2 Cycle	− 34.1%	13.87	150.71
15	YCLO-63	PD	2 Cycle	2 Cycle	+ 20.7%	137.02	882.76

G/A, gemcitabine plus nab-paclitaxel (abraxane); CRC, conditional reprogrammed cell; YCLO, YPAC cell line organoid; PD, progressive disease; SD, stable disease; PR, partial response; IC50; Inhibitory concentration 50

**Table 2:** Overview of the first FOLFIRINOX (FFX) response in established CRC cell line 2D and organoid culture conditions

No	Samples	Best Response	Total Chemotherapy Cycles	Chemotherapy Cycles until Best Response	Tumor Size Change	2D IC50 (µM)	Organoid IC50 (µM)
1	YCLO-2	PD	4 Cycle	4 Cycle	0.0%	29.19	160.33
2	YCLO-18	PR	13 Cycle	8 Cycle	− 27.4%	253.67	255.58
3	YCLO-21	SD	20 Cycle	4 Cycle	+ 6.7%	62.42	33.81
4	YCLO-26	SD	9 Cycle	4 Cycle	+ 9.8%	50.12	32.14
5	YCLO-27	PR	15 Cycle	4 Cycle	− 30.1%	71.99	197.56
6	YCLO-62	SD	40 Cycle	4 Cycle	− 14.8%	207.42	189.08
7	YCLO-35	SD	9 Cycle	4 Cycle	− 6.5%	28.14	23.95
8	YCLO-38	PD	3 Cycle	3 Cycle	+ 15.1%	193.36	275.01
9	YCLO-39	PD	4 Cycle	4 Cycle	+ 0.2%	127.87	222.33
10	YCLO-40	PR	14 Cycle	4 Cycle	− 60.9%	25.34	19.73
11	YCLO-47	SD	20 Cycle	4 Cycle	− 13.9%	39.98	197.84
12	YCLO-50	PD	4 Cycle	4 Cycle	0.0%	20.03	238.19
13	YCLO-51	SD	10 Cycle	4 Cycle	− 16.2%	146.24	151.81
14	YCLO-54	SD	8 Cycle	4 Cycle	− 17.3%	9.49	8.51
15	YCLO-60	PR	17 Cycle	4 Cycle	− 31.4%	16.53	22.89
16	YCLO-64	PR	34 Cycle	4 Cycle	-32.4%	24.97	142.11
17	YCLO-65	PR	19 Cycle	4 Cycle	-33.2%	41.10	271.42

FFX; FOLFIRINOX; CRC, conditional reprogrammed cell; YCLO, YPAC cell line organoid; PD, progressive disease; SD, stable disease; PR, partial response; IC50; Inhibitory concentration 50

Additionally, we analyzed the area under the curve (AUC) values to compare the predictive performance between PD and PR/SD groups. In the G/A dataset, the 3D CRC organoid model demonstrated superior classification performance (AUC = 0.839, *p* = 0.022) compared to the 2D CRC model (AUC = 0.651, *p* = 0.681) (Supplementary Fig. S9A). Similarly, in the FFX dataset, the organoid model exhibited higher predictive accuracy (AUC = 0.827, *p* = 0.014), whereas the 2D CRC model showed lower predictive power (AUC = 0.645, *p* = 0.725) (Supplementary Fig. S9B). Subsequently, the association between drug sensitivity and organoid type or clinical parameters was analyzed, but no significant correlations were observed (Supplementary Fig. S10 and S11). This result aligns with clinical observations, where drug sensitivity does not appear to be associated with these clinical parameters.

## Discussion

In this study, we established a 3D organoid culture platform using patient-derived 2D CRC cells. The CRC organoids retained the molecular characteristics and key oncogenic and tumor suppressor gene mutations of the original tumors. A comprehensive analysis of mutational and transcriptomic profiles across matched tumor tissues, CRCs, and corresponding organoids confirmed that the established organoids maintain a high level of genetic and transcriptomic concordance with their parental tumor tissues. Furthermore, CRC organoids exhibited distinct morphological features that corresponded to the cancer stages and cell differentiation observed in patients. Notably, the drug response profiles of CRC organoids to FOLFIRINOX and G/A closely reflected the clinical responses of patients, demonstrating greater predictive accuracy than 2D CRC cells. We observed that the established CRC organoids, when digested and reseeded in 2D culture, retained their original morphological characteristics and exhibited robust growth. This indicates that the platform we developed supports a flexible, bidirectional transition between two-dimensional (2D) and three-dimensional (3D) culture conditions, adaptable to experimental needs. These findings highlight the utility of CRC organoid models as robust preclinical tools for exploring the molecular biology of pancreatic cancer and evaluating therapeutic agents.

In this study, we aimed to establish CRC organoids using 86 pre-established patient-derived CRCs, successfully generating organoids in 66 cases (success rate: 77%). While no significant differences were observed in clinical features or molecular characteristics between the success and failure groups, it is noteworthy that all 20 CRCs in which organoid establishment was unsuccessful were KRAS-wild type. Specifically, organoids were successfully established from all 63 KRAS-mutant CRCs (100% success rate), whereas only 3 of the 23 KRAS-wild type CRCs (13% success rate) resulted in successful organoid establishment. KRAS mutations are known to promote cell survival, proliferation, and metabolic activity by constitutively activating downstream signaling pathways, particularly the MAPK/ERK and PI3K/AKT pathways [35–38]. We think that these oncogenic features may confer a growth advantage to KRAS-mutant cancer cells under 3D culture conditions, especially in our system, where additional growth factors such as Wnt3a, R-spondin-1, and Noggin were excluded. Given that KRAS mutations are detected in approximately 95% of pancreatic cancers, we believe the organoid establishment process utilized in our study would be broadly applicable to the majority of pancreatic cancer cases.

Previously, patient-derived CRCs were established from tumor samples of patients with PDAC under 2D culture conditions. These CRC cell lines, characterized by their genetic and molecular subtypes, in vitro therapeutic profiles, in vivo tumorigenesis, and drug sensitivity, served as unique ex vivo models for personalized PDAC therapies. However, several limitations were associated with these CRC cell lines [39–42]. While single-agent responses in 2D CRCs aligned with clinical observations, discrepancies arose when comparing drug responses in 2D CRCs with those observed in patients treated with drug combinations typical of clinical practice. In this study, patient-derived CRCs were advanced into a 3D organoid model. Specifically, 3D CRC organoids demonstrated drug responses consistent with those of patients treated with G/A or FOLFIRINOX at clinically relevant ratios. Given the absence of specific biomarkers for predicting drug responses in pancreatic cancer, this 3D CRC organoid model represents a valuable tool for preclinical validation of drug efficacy before patient administration.

Pancreatic cancer is categorized into two primary molecular subtypes based on transcriptome analysis [43–47]. Notably, responsiveness to therapeutic agents differs between these molecular subtypes [43, 46–48]. Single-cell transcriptome analysis has revealed that pancreatic cancer comprises a heterogeneous population of cancer cells with distinct subtypes, indicating that the predominant subtype in a patient’s tumor influences the response to anticancer drugs [49–52]. Previous studies have reported that pancreatic cancer organoids gradually shift toward the classical subtype as passages increase, attributed to growth factors present in organoid culture media, such as Wnt3a, R-spondin-1, and Noggin, which play a crucial role in maintaining stemness and promoting proliferation [20–25]. Consequently, changes in cancer cell subtypes during organoid culture may modify responsiveness to anticancer drugs over time. In this study, we developed CRC organoids using pre-established CRCs that had already acquired stemness and proliferative capacity during their establishment. Therefore, additional components such as Wnt3a, R-spondin-1, and Noggin, which are known to influence subtype transitions during organoid formation and maintenance were intentionally excluded from our culture media. This approach was designed to minimize potential subtype alterations during both the establishment and long-term maintenance of organoids. By stabilizing organoid characteristics, we anticipated that this method would yield more reliable drug screening results.

In this study, we observed that organoids could develop into distinct morphological forms depending on their differentiation status, even when cultured under identical conditions. Previous reports have indicated that normal pancreatic organoids typically exhibit a cystic shape; interestingly, some tumor-derived organoids in our study also demonstrated this morphology. Since the organoids in this study were not directly derived from primary tumor tissues but instead from validated cancer cell lines (CRCs), it is highly unlikely that the cystic organoids resulted from normal cell contamination. To confirm this, we isolated cystic-shaped organoids and performed mutation profiling, which revealed identical mutations to those found in the parental tumor tissues (Data not shown). For certain organoids displaying both cystic and compact structural features, further studies will be necessary to determine whether one morphological subtype becomes dominant over prolonged culture.

Currently, one of the major limitations in establishing a high-throughput drug screening platform using 3D organoid models is their high cost and the difficulty of eliminating normal cell contamination. Additionally, repeated subculture can lead to molecular feature alterations due to various components in the organoid culture medium such as Wnt3a, R-spondin-1, and Noggin, which we intentionally excluded from our model [53, 54]. In this study, we first established patient-derived pancreatic cancer CRCs under 2D conditions and subsequently used them for organoid culture. This approach enables straightforward assessment of molecular features and mutation profiles during the 2D cell line establishment process, while also facilitating the removal of normal cell contamination. Moreover, cells can be expanded and maintained long-term under 2D conditions in a cost-effective manner without altering their molecular subtype. When needed, for instance, drug screening assays, these cells can be readily transitioned into 3D organoids, which better reflect patients’ clinical responses than 2D cells. They can then return to 2D culture for continued maintenance in a stable and cost-effective manner. Recently, Takeuchi et al. [55] suggested a fused pancreatic cancer organoid model by co-culturing pancreatic cancer organoids with human-induced pluripotent stem cell (hiPSC)-derived endothelial and mesenchymal cells to better mimic the tumor microenvironment. While incorporating diverse components of the tumor microenvironment can enhance structural similarity to in vivo tumors, our recent single-cell transcriptomic analysis of 17 tumor tissues of pancreatic cancer revealed significant variability in stromal cell composition across individuals [52]. Therefore, co-culturing patient-specific organoids with general (non-patient-specific) endothelial and mesenchymal cells may create an artificial microenvironment that diverges from the actual tumor biology of each patient. Given that stromal-tumor interactions can influence drug sensitivity, models using stromal cells that differ from those of the actual patient may produce drug screening results that deviate from clinical outcomes. From this perspective, we believe the organoids established in our study provide a more reliable platform for drug screening, at least under current methodologies.

## Conclusions

The findings of this study highlight the utility of patient-derived 3D CRC organoid models as a bridge between in vitro drug screening and clinical outcomes. By closely replicating the three-dimensional tumor structure and preserving the molecular and mutational characteristics of primary tumors, these organoids provide a physiologically relevant platform for evaluating drug efficacy. Notably, the ability of 3D organoids to reflect patient-specific drug responses underscores their potential in precision oncology, particularly for pancreatic cancer, where the absence of predictive biomarkers limits effective treatment selection. Beyond drug screening, these organoid models can facilitate the identification of novel therapeutic targets and the development of combination therapies by enabling a deeper understanding of tumor heterogeneity and resistance mechanisms. Despite these advantages, the practical limitations of 3D organoid systems, including cost, time requirements, and scalability for large-scale studies, remain challenges that require further optimization. Future efforts to standardize organoid culture conditions and integrate automated technologies could enhance their applicability in preclinical and clinical settings.

## Electronic supplementary material

Below is the link to the electronic supplementary material.

Supplementary Material 1

Supplementary Material 2

Supplementary Material 3

Supplementary Material 4

Supplementary Material 5

## Data Availability

RNA sequencing data have been deposited in the Gene Expression Omnibus (GEO) database under accession number GSE297770. Targeted deep sequencing data are available for download through the Sequence Read Archive (SRA) under BioProject ID: PRJNA1265782. All other supporting data and materials related to this study can be obtained from the corresponding author, GL, upon reasonable request.

## Abbreviations

BFM: Bright-field microscopy
CRC: Conditional reprogramming cell
DNA: Deoxyribonucleic acid
DAPI: 4’,6-diamidino-2-phenylindole
EGF: Epidermal growth factor
FOLFIRINOX: 5-fluorouracil, folinic acid, irinotecan, and oxaliplatin
G/A: Gemcitabine plus nab-paclitaxel (Abraxane)
IC50: Half maximal inhibitory concentration
IF: Immunofluorescence
KRAS: Kirsten rat sarcoma virus oncogene homolog
OsO4: Osmium tetroxide
PBS: Phosphate-buffered saline
PFA: Paraformaldehyde
PCR: Polymerase chain reaction
PDAC: Pancreatic ductal adenocarcinoma
RNA: Ribonucleic acid
SEM: Scanning electron microscopy
SMAD4: Mothers against decapentaplegic homolog 4
ULGT: Ultra-low-gelling temperature
