# Supplementary material for: Establishing 3D organoid models from patient-derived conditionally reprogrammed cells to bridge preclinical and clinical insights in pancreatic cancer
Source: Mol Cancer. 2025 Jun 3;24:162. doi: 10.1186/s12943-025-02374-y (PMC12131615; doi:10.1186/s12943-025-02374-y)
Supplement: Supplementary file 1 — Supplementary Material 1 [file 12943_2025_2374_MOESM1_ESM.pdf]

Supplementary Figure S1

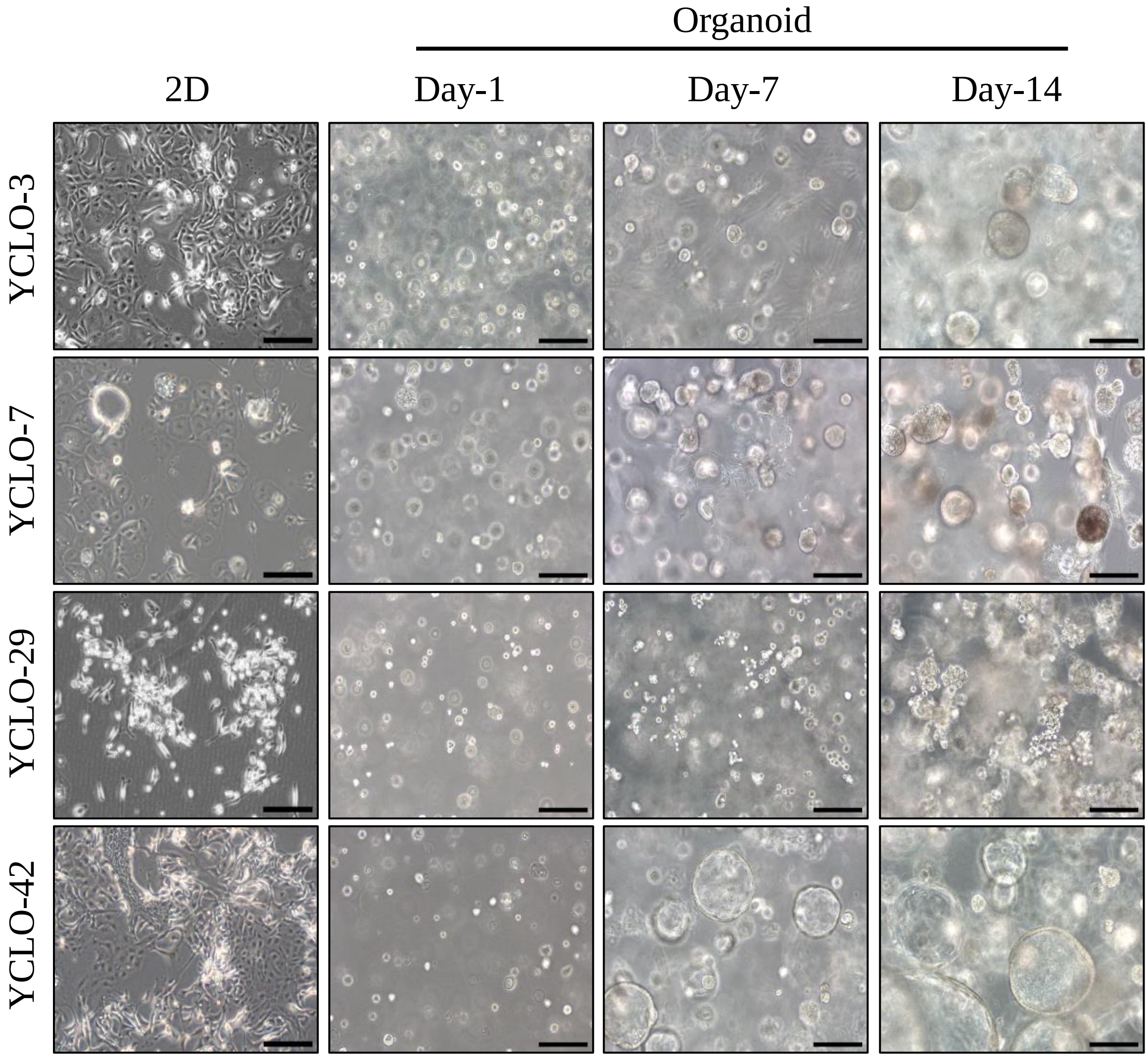

Supplementary Figure S1. Serial morphological observation of CRC organoid formation. Bright-field microscopy (BFM) images of representative CRC cell lines (YCLO-3, YCLO-7, YCLO-29, YCLO-42) showing the transition from 2D cultures to organoid structures over 14 days. Organoids typically began to form within 1 week after cell seeding and exhibited various morphologies, including compact and cystic structures. Scale bar: 200 μm.

Supplementary Figure S2

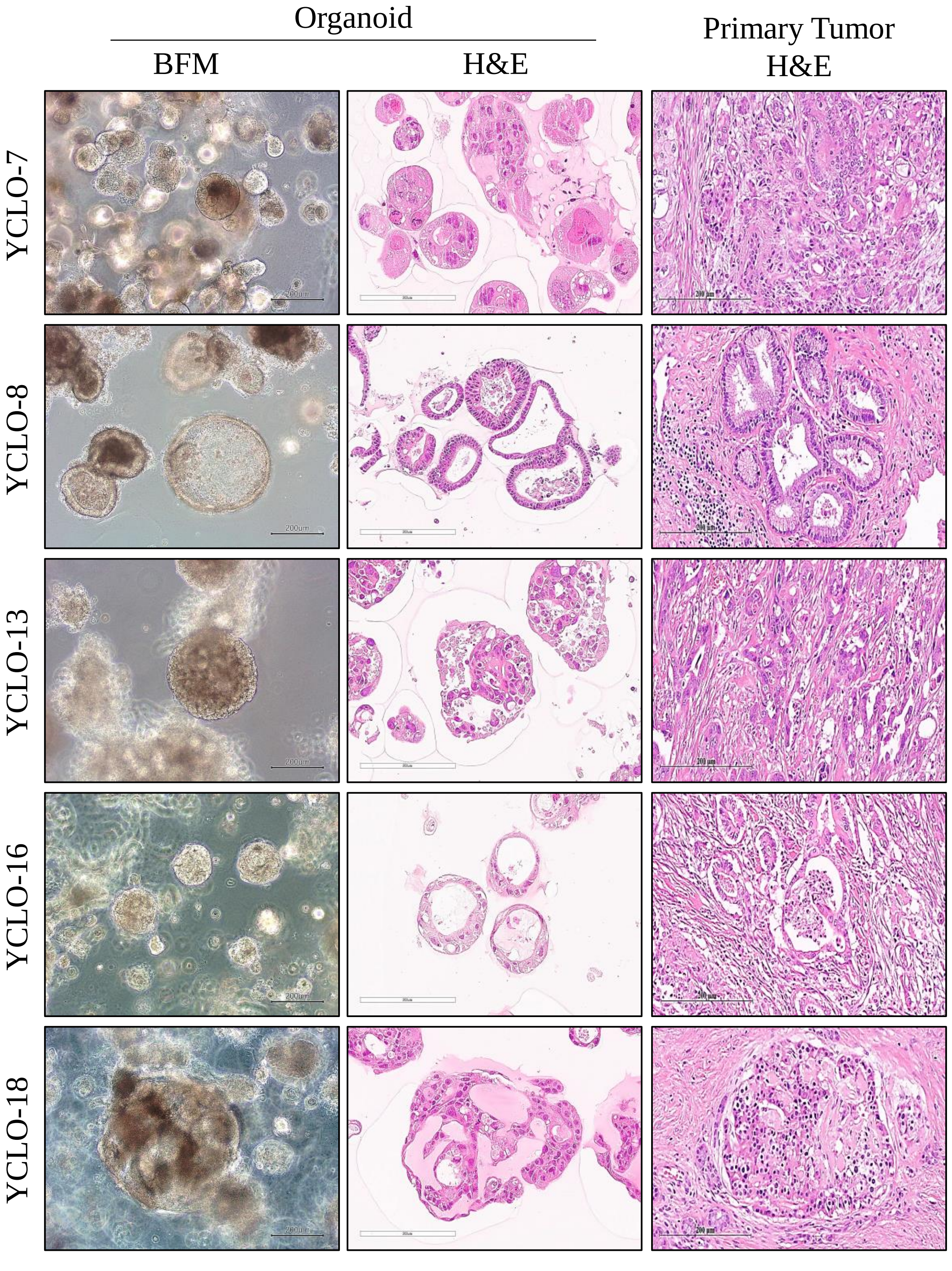

Supplementary Figure S2. Histological comparison between established CRC organoids and matched primary tumor tissues. Bright-field microscopy (BFM) and hematoxylin and eosin (H&E) staining of representative CRC organoids (YCLO-7, YCLO-8, YCLO-13, YCLO-16, YCLO-18) are shown alongside H&E-stained sections of the corresponding primary pancreatic tumor tissues. The morphological and histological features of the CRC organoids were well preserved, reflecting the architecture of their matched primary tumors. Scale bar: 200 μm.

Supplementary Figure S3

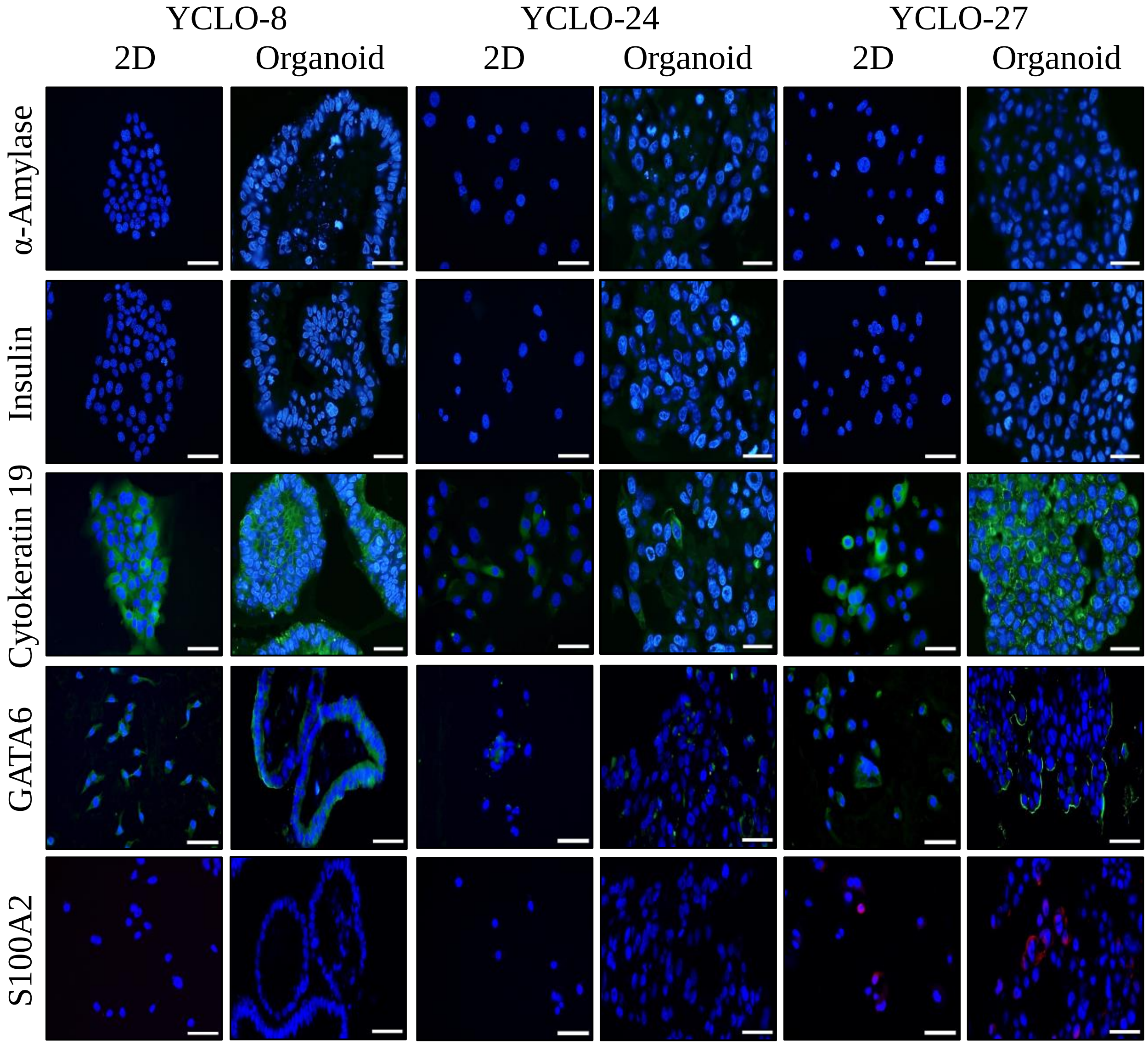

Supplementary Figure S3. Immunofluorescence analysis of CRC 2D and organoid cultures. Immunofluorescence (IF) staining was performed on established CRC cell line 2D and organoid samples (YCLO-8, YCLO-24, YCLO-27) using markers for ductal epithelial cells (cytokeratin-19; CK-19), acinar cells (α-amylase), islet cells (insulin), the classical subtype marker GATA6, and the basal subtype marker S100A2. CK-19 was consistently expressed in both 2D and organoid cultures. In contrast, α-amylase and insulin were not detected. While the expression levels of GATA6 and S100A2 varied across samples, each marker showed a consistent staining pattern between 2D and corresponding organoid cultures, indicating that subtype-specific features of the original cells were preserved during the transition from 2D to 3D culture. Scale bar: 50 μm.

Supplementary Figure S4

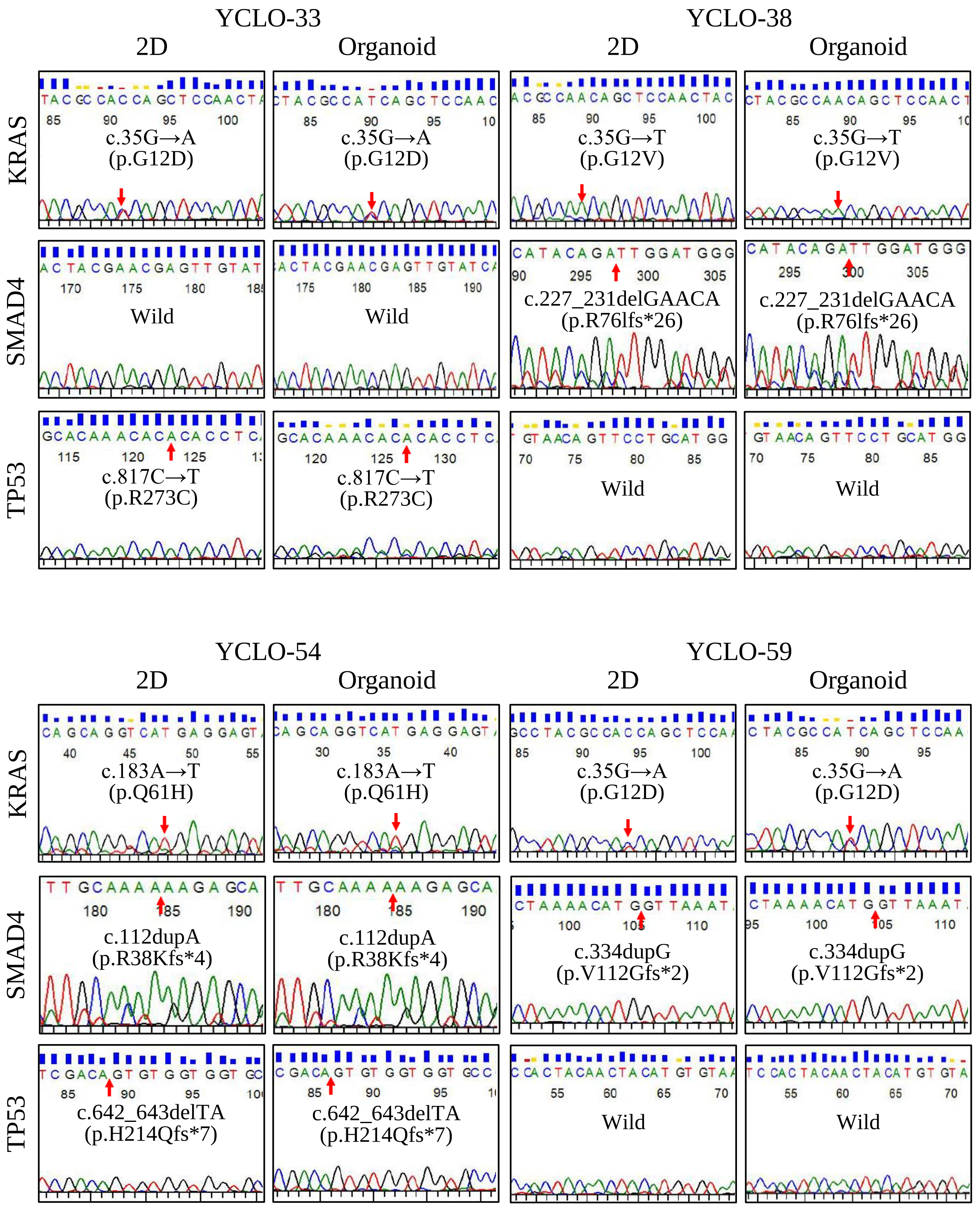

Supplementary Figure S4. KRAS, SMAD4 and TP53 mutation status in established CRC cell lines under 2D and organoid culture conditions. For genomic regions where mutations were reported in the patient's NGS data, PCR amplification and Sanger sequencing were performed using matched 2D and organoid culture conditions. The results showed complete concordance between the two culture conditions. Red arrows indicate the sites of confirmed mutations.

Supplementary Figure S5

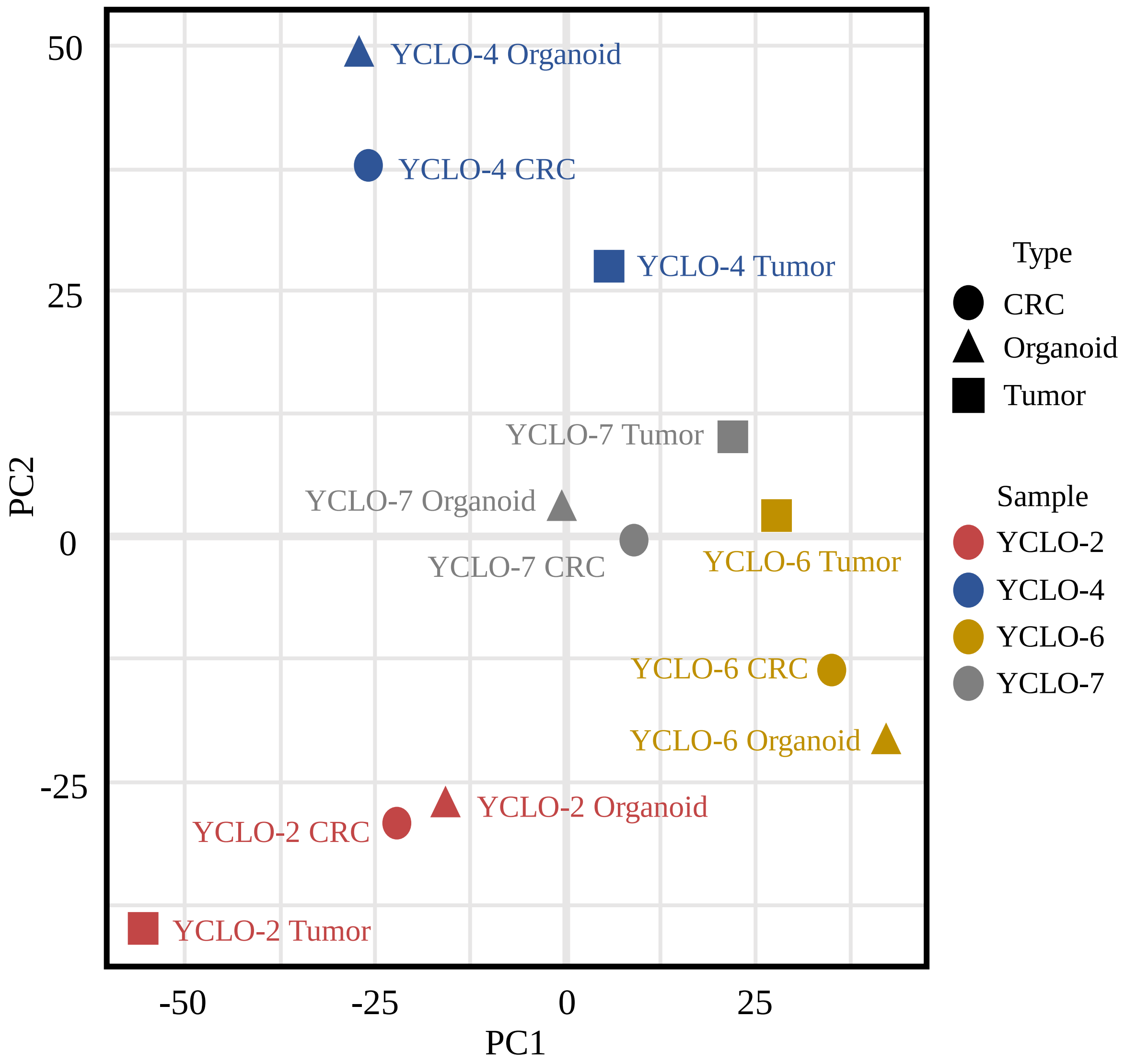

Supplementary Figure S5. Principal Component Analysis (PCA) of CRC cells, organoids, and matched tumor tissues. Principal Component Analysis (PCA) of transcriptomic profiles from matched primary tumors CRCs, and organoid. This dimensionality reduction approach was used to visualize and assess the global transcriptomic similarity among sample types. Each point represents an individual sample, with shapes indicating sample type (square: tumor, circle: CRC, triangle: organoid) and colors denoting patient identity (red: YCLO-2, blue: YCLO-4, yellow: YCLO-6, gray: YCLO-7). Samples clustered primarily by patient of origin, rather than sample type, indicating that both CRCs and organoids retain transcriptomic profiles closely aligned with their original tumor tissue.

Supplementary Figure S6

A

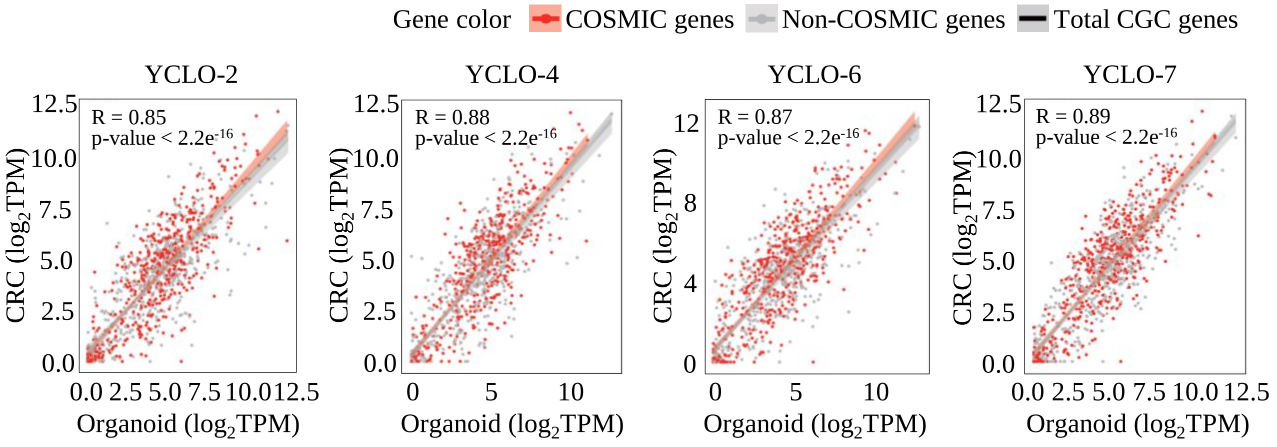

B

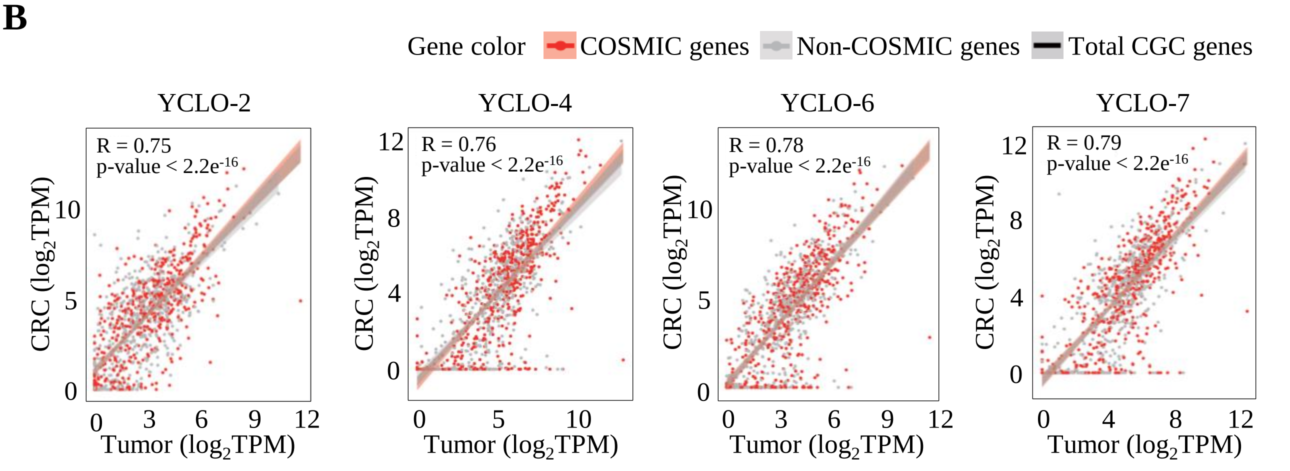

Supplementary Figure S6. Pairwise correlation of cancer-related gene expression demonstrates high concordance between CRCs, organoids, and tumor. Correlation between gene expression profiles of tumor, CRC, and organoid across four samples. **(A)** Each scatter plot shows the $\log_2$-transformed TPM (Transcripts Per Million) values of CGC genes between matched CRC and organoid samples for each patient. Red dots represent genes listed in the COSMIC database, while grey dots represent non-COSMIC genes among the CGC gene set. The black regression line represents the linear fit based on all CGC genes, with the shaded area indicating the 95% confidence interval. Pearson correlation coefficients (R) and associated p-values are reported in each panel. COSMIC genes demonstrate strong concordance between tumor and organoid expression, indicating a strong concordance between tumor and organoid expression profiles for cancer-relevant genes. **(B)** Scatter plots show the correlation of CGC gene expression between matched CRC and Tumor tissue samples from the same patients. Plotting conventions are identical to those described in panel **(A)**, allowing direct comparison of gene expression concordance between primary tissue and derived models.

Supplementary Figure S7

A

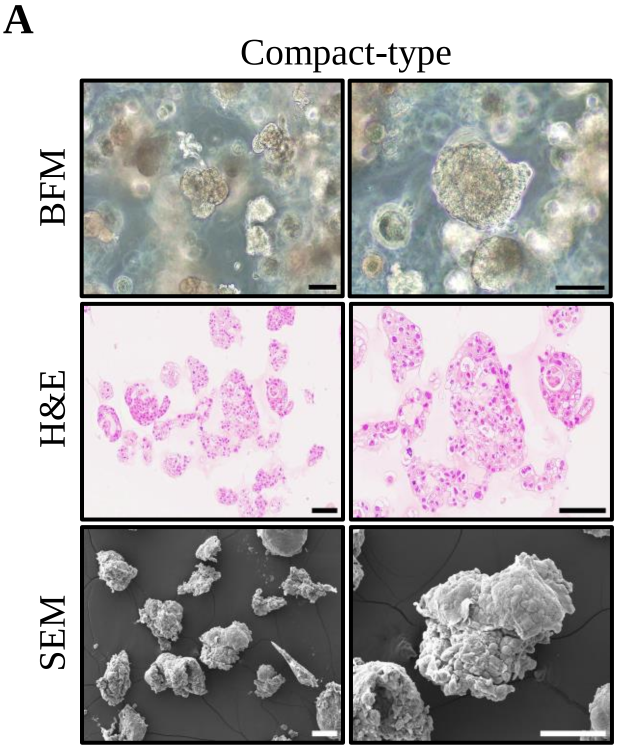

B

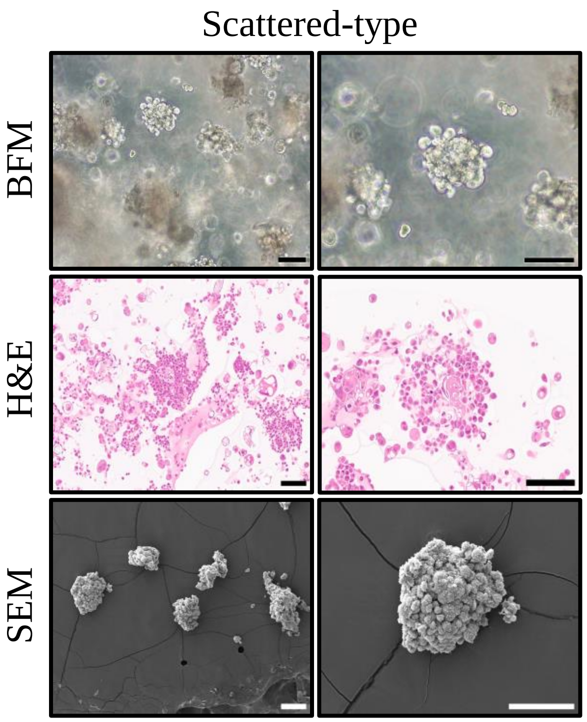

C

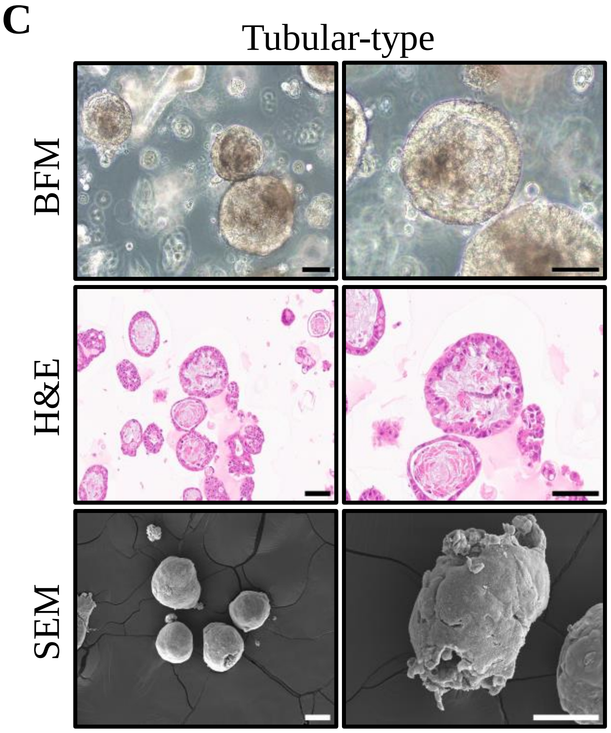

Supplementary Figure S7. Representative morphological images of compact, scattered, and tubular CRC organoid types using BFM, H&E staining, and SEM. CRC organoids exhibited three distinct morphological subtypes. **(A)** Compact-type organoids showed dense, solid structures. **(B)** Scattered-type organoids presented loosely connected or separated cells. **(C)** Tubular-type organoids displayed hollow, duct-like luminal structures. Morphological features were consistently observed across bright-field microscopy (BFM), hematoxylin and eosin (H&E) staining, and scanning electron microscopy (SEM) images. Scale bar: 100 µm.

Supplementary Figure S8

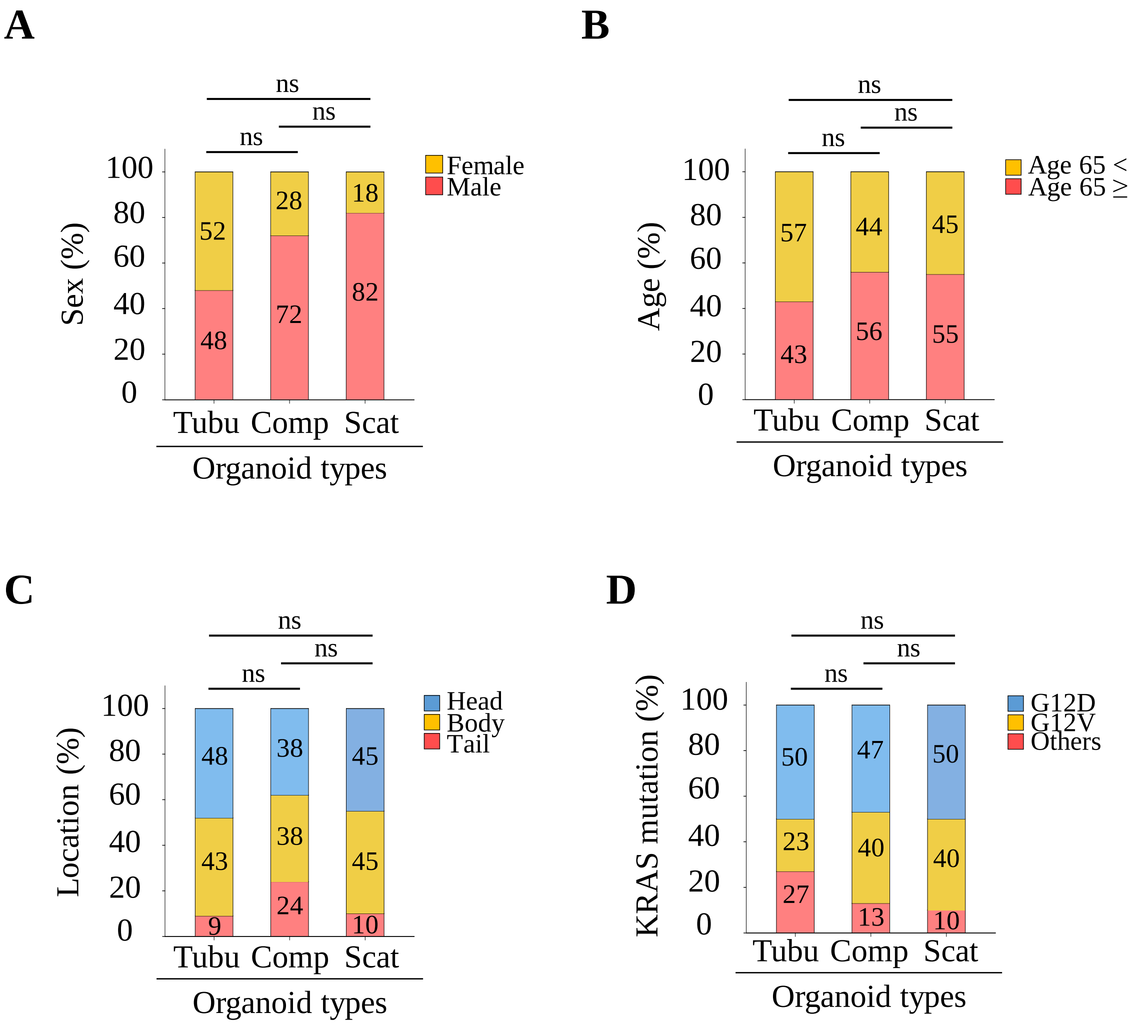

Supplementary Figure S8. Distribution of established CRC organoid types based on clinical parameters of pancreatic cancer patients. (A–D) CRC organoids were classified into tubular, compact, and scattered types and analyzed for correlation with patients' sex **(A)**, age **(B)**, tumor location within the pancreas **(C)**, and KRAS mutation subtype **(D)**. No significant associations were observed between organoid morphological types and clinical parameters. Statistical analysis: Fisher's exact test. ns, not significant.

Supplementary Figure S9

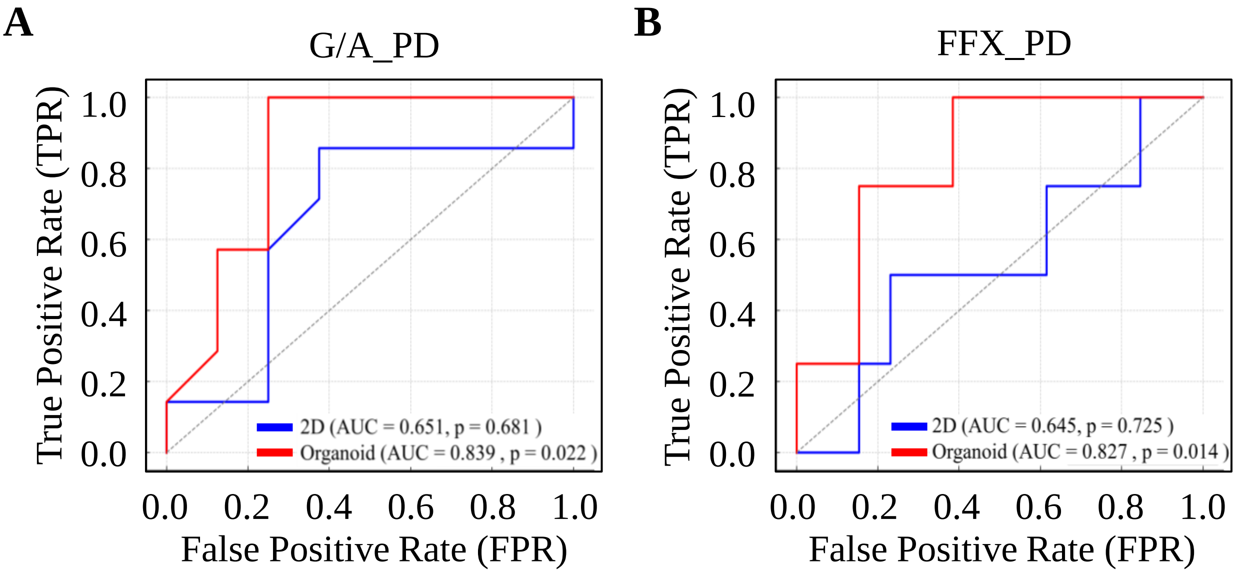

Supplementary Figure S9. AUC analysis of IC50-based prediction in 2D and organoid cultures. ROC curves compare the predictive performance of IC50 values in 2D and organoid cultures for distinguishing PD from SD/PR. Organoid cultures (G/A: AUC = 0.839, p = 0.022; FFX: AUC = 0.827, p = 0.014) showed superior predictive accuracy compared to 2D cultures (G/A: AUC = 0.651, p = 0.681; FFX: AUC = 0.645, p = 0.725). These results suggest that organoid-based drug testing more effectively predicts PD status in pancreatic cancer patients. Abbreviations: AUC, area under the curve; ROC, receiver operating characteristic; G/A, Gemcitabine plus nab-paclitaxel (Abraxane); FFX, FOLFIRINOX; IC50, half maximal inhibitory concentration; PR, partial response; SD, stable disease; PD, progressive disease.

Supplementary Figure S10

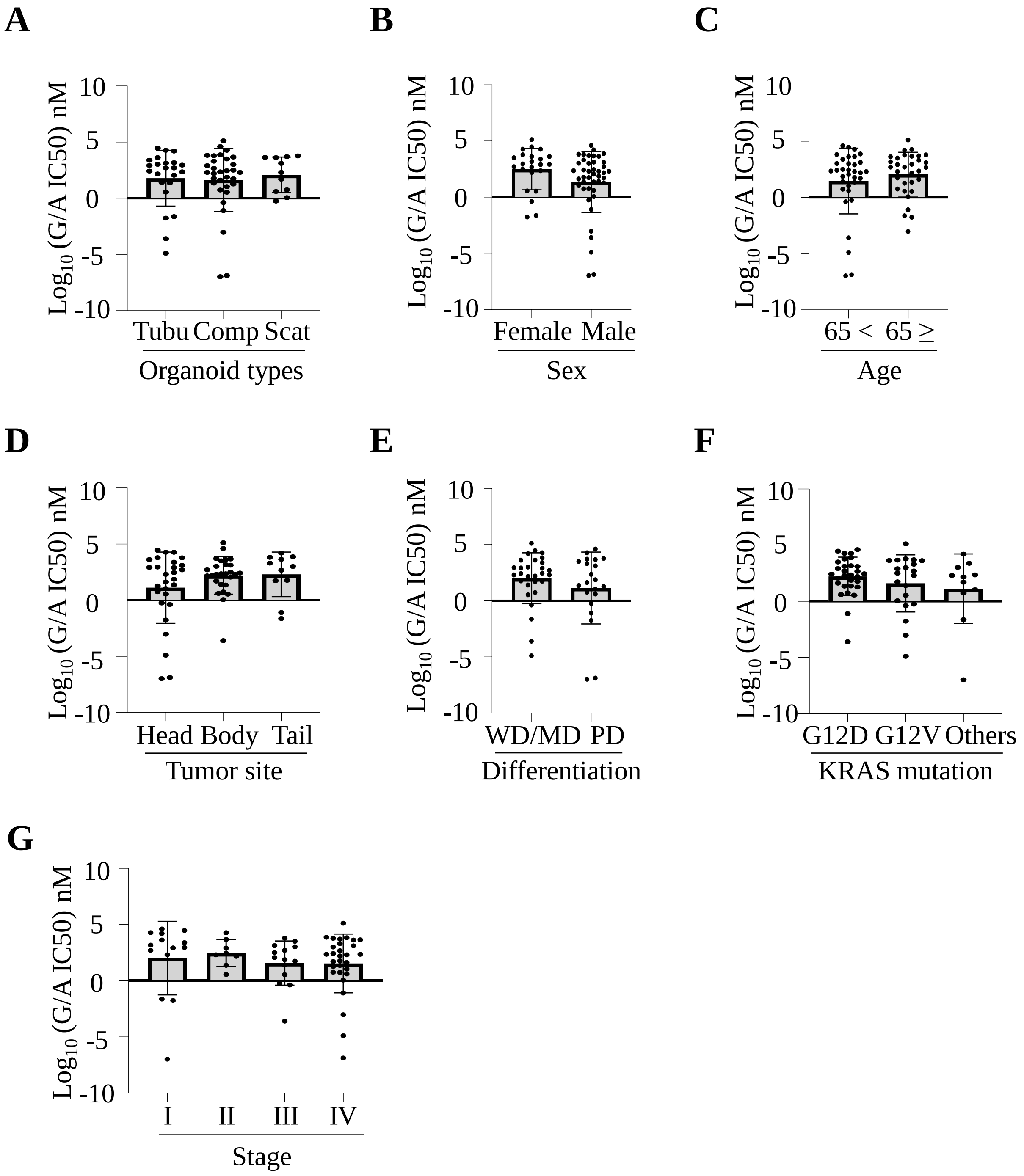

Supplementary Figure S10. Prediction of Gemcitabine plus nab-paclitaxel (Abraxane) (G/A) sensitivity in CRC organoids according to organoid types and clinical features. (A–G) Under 3D organoid culture conditions, G/A sensitivity (Log_{10} IC50 values) was compared based on organoid morphological types (Tubular, Compact and Scattered) **(A)**, patient sex **(B)**, age **(C)**, tumor location **(D)**, tumor differentiation status **(E)**, KRAS mutation subtype **(F)**, and cancer stage **(G)**. No significant associations were observed. For statistical analysis, clinical stages were grouped as follows: Stage I included 1, 1A, and 1B; Stage II included 2, 2A, and 2B. Statistical analysis: Kruskal-Wallis test or Mann-Whitney U test. Abbreviations: CRC, conditionally reprogrammed cells; IC50, half-maximal inhibitory concentration; WD, well-differentiated; MD, moderately differentiated; PD, poorly differentiated.

Supplementary Figure S11

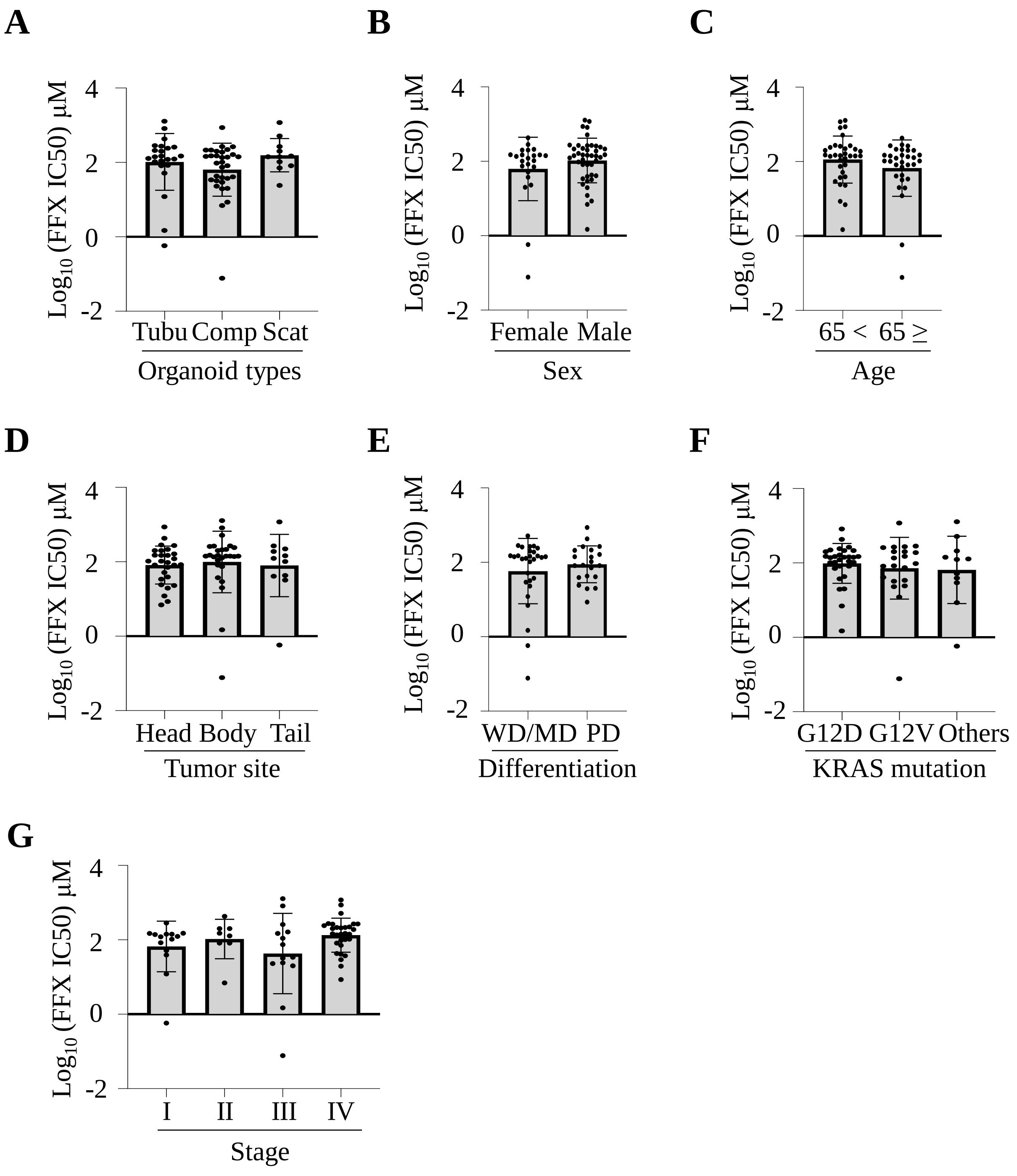

Supplementary Figure S11. Prediction of FOLFIRINOX sensitivity in CRC organoids according to organoid types and clinical features. (A–G) Under 3D organoid culture conditions, FOLFIRINOX sensitivity (Log_{10} IC50 values) was compared based on organoid morphological types (Tubular, Compact and Scattered) **(A)**, patient sex **(B)**, age **(C)**, tumor location **(D)**, tumor differentiation status **(E)**, KRAS mutation subtype **(F)**, and cancer stage **(G)**. No significant associations were observed. For statistical analysis, clinical stages were grouped as follows: Stage I included 1, 1A, and 1B; Stage II included 2, 2A, and 2B. Statistical analysis: Kruskal-Wallis test or Mann-Whitney U test. Abbreviations: CRC, conditionally reprogrammed cells; IC50, half-maximal inhibitory concentration; WD, well-differentiated; MD, moderately differentiated; PD, poorly differentiated.
