# Supplementary material for: Establishing 3D organoid models from patient-derived conditionally reprogrammed cells to bridge preclinical and clinical insights in pancreatic cancer
Source: Mol Cancer. 2025 Jun 3;24:162. doi: 10.1186/s12943-025-02374-y (PMC12131615; doi:10.1186/s12943-025-02374-y)
Supplement: Supplementary file 2 — Supplementary Material 2 [file 12943_2025_2374_MOESM2_ESM.docx]

**Supplementary Table S1. Inclusion and exclusion criteria of this study**

| Inclusion criteria |
| --- |
| 1. Age ≥ 20 2. A patient newly diagnosed with pancreatic cancer based on imaging studies (CT or MRI) 3. A patient who voluntarily agreed to participate in this study |
| Exclusion criteria |
| 1. A patient with a history of being diagnosed and treated for another cancer within the past five years 2. A patient for whom tissue biopsy via endoscopic ultrasound(EUS)-guided biopsy is not feasible |
