# Supplementary material for: Establishing 3D organoid models from patient-derived conditionally reprogrammed cells to bridge preclinical and clinical insights in pancreatic cancer
Source: Mol Cancer. 2025 Jun 3;24:162. doi: 10.1186/s12943-025-02374-y (PMC12131615; doi:10.1186/s12943-025-02374-y)
Supplement: Supplementary file 3 — Supplementary Material 3 [file 12943_2025_2374_MOESM3_ESM.docx]

**Supplementary Table S2. Targeted Deep Sequencing Panels.**

| **348 Gene Lists** | | | | | | | | | |
| --- | --- | --- | --- | --- | --- | --- | --- | --- | --- |
| ABL1 | ACVR1B | AKT1 | AKT2 | AKT3 | ALK | ALOX12B | AMER1 | APC | AR |
| ARAF | ARFRP1 | ARID1A | ARID2 | ASXL1 | ATM | ATR | ATRX | AURKA | AURKB |
| AXIN1 | AXL | BAP1 | BARD1 | BCL2 | BCL2L1 | BCL2L2 | BCL6 | BCOR | BCORL1 |
| BCR | BRAF | BRCA1 | BRCA2 | BRD4 | BRIP1 | BTG1 | BTG2 | BTK | C11ORF30 |
| CALR | CARD11 | CASP8 | CBFB | CBL | CCND1 | CCND2 | CCND3 | CCNE1 | CD22 |
| CD274 | CD70 | CD74 | CD79A | CD79B | CDC73 | CDH1 | CDK12 | CDK4 | CDK6 |
| CDK8 | CDKN1A | CDKN1B | CDKN2A | CDKN2B | CDKN2C | CEBPA | CHEK1 | CHEK2 | CIC |
| CREBBP | CRKL | CRLF2 | CSF1R | CSF3R | CTCF | CTNNA1 | CTNNB1 | CUL3 | CUL4A |
| CXCR4 | CYP17A1 | DAXX | DDR1 | DDR2 | DICER1 | DIS3 | DNMT1 | DNMT3A | DOT1L |
| ABL1 | ACVR1B | AKT1 | AKT2 | AKT3 | ALK | ALOX12B | AMER1 | APC | AR |
| EED | EGFR | EP300 | EPHA3 | EPHB1 | EPHB4 | ERBB2 | ERBB3 | ERBB4 | ERCC4 |
| ERG | ERRFI1 | ESR1 | ETV1 | ETV5 | ETV6 | EWSR1 | EZH2 | EZR | FAM46C |
| FANCA | FANCC | FANCG | FANCL | FAS | FBXW7 | FGF10 | FGF12 | FGF14 | FGF19 |
| FGF23 | FGF3 | FGF4 | FGF6 | FGFR1 | FGFR2 | FGFR3 | FGFR4 | FH | FLCN |
| FLT1 | FLT3 | FOXL2 | FUBP1 | GABRA6 | GATA1 | GATA2 | GATA3 | GATA4 | GATA6 |
| GID4 | GNA11 | GNA13 | GNAQ | GNAS | GRIN2A | GRM3 | GSK3B | H3F3A | HDAC1 |
| HGF | HIST1H3B | HNF1A | HRAS | HSD3B1 | ID3 | IDH1 | IDH2 | IGF1R | IKBKE |
| IKZF1 | IL7R | INPP4B | IRF2 | IRF4 | IRS2 | JAK1 | JAK2 | JAK3 | JUN |
| KDM5A | KDM5C | KDM6A | KDR | KEAP1 | KEL | KIT | KLHL6 | KMT2A | KMT2C |
| KMT2D | KRAS | LTK | LYN | MAF | MAP2K1 | MAP2K2 | MAP2K4 | MAP3K1 | MAP3K13 |
| MAPK1 | MCL1 | MDM2 | MDM4 | MED12 | MEF2B | MEN1 | MERTK | MET | MITF |
| MKNK1 | MLH1 | MPL | MRE11A | MSH2 | MSH3 | MSH6 | MST1R | MTAP | MTOR |
| MUTYH | MYC | MYCL | MYCN | MYD88 | NBN | NF1 | NF2 | NFE2L2 | NFKBIA |
| NKX2-1 | NOTCH1 | NOTCH2 | NOTCH3 | NPM1 | NRAS | NT5C2 | NTRK1 | NTRK2 | NTRK3 |
| P2RY8 | PAK7 | PALB2 | PARK2 | PARP1 | PARP2 | PARP3 | PAX5 | PBRM1 | PDCD1 |
| PDCD1LG2 | PDGFRA | PDGFRB | PDK1 | PIK3C2B | PIK3C2G | PIK3CA | PIK3CB | PIK3R1 | PIM1 |
| PMS2 | POLD1 | POLE | PPARG | PPP2R1A | PPP2R2A | PRDM1 | PREX2 | PRKAR1A | PRKCI |
| PTCH1 | PTEN | PTPN11 | PTPRD | PTPRO | QKI | RAC1 | RAD21 | RAD51 | RAD51B |
| RAD51C | RAD51D | RAD52 | RAD54L | RAF1 | RARA | RB1 | RBM10 | REL | RET |
| RHOA | RICTOR | RIT1 | RNF43 | ROS1 | RPTOR | RUNX1 | SDHA | SDHB | SDHC |
| SDHD | SETD2 | SF3B1 | SGK1 | SMAD2 | SMAD4 | SMARCA4 | SMARCB1 | SMO | SNCAIP |
| SOCS1 | SOX2 | SOX9 | SPEN | SPOP | SRC | SRSF2 | STAG2 | STAT3 | STK11 |
| SUFU | SYK | TBX3 | TEK | TERC | TERT | TET2 | TGFBR2 | TIPARP | TMPRSS2 |
| TNFAIP3 | TNFRSF14 | TP53 | TP63 | TSC1 | TSC2 | TSHR | TYRO3 | U2AF1 | VEGFA |
| VHL | WHSC1 | WHSC1L1 | WT1 | XPO1 | XRCC2 | ZNF217 | ZNF703 |  |  |
|  | | | | | | | | | |
