# Supplementary material for: Establishing 3D organoid models from patient-derived conditionally reprogrammed cells to bridge preclinical and clinical insights in pancreatic cancer
Source: Mol Cancer. 2025 Jun 3;24:162. doi: 10.1186/s12943-025-02374-y (PMC12131615; doi:10.1186/s12943-025-02374-y)
Supplement: Supplementary file 4 — Supplementary Material 4 [file 12943_2025_2374_MOESM4_ESM.docx]

**Supplementary Table S3. Reagent and resource lists.**

| Reagent or resource | Source | Identifier |
| --- | --- | --- |
|  | | |
| Antibodies | | |
| α-Amylase rabbit polyclonal antibody | Santa Cruz Biotechnology | Catalog no. sc-25562 |
| Cytokeratin 19 mouse monoclonal antibody | Santa Cruz Biotechnology | Catalog no. sc-6278 |
| Insulin mouse monoclonal antibody | Santa Cruz Biotechnology | Catalog no. sc-8033 |
| Vimentin rabbit monoclonal antibody | Cell Signaling Technology | Catalog no. D21H3 |
| GATA6 mouse monoclonal antibody | Invitrogen | Catalog no. MA5-38529 |
| S100A2 rabbit monoclonal antibody | Abcam | Catalog no. ab109494 |
| Alexa Fluor 488 mouse secondary antibody | Invitrogen | Catalog no. A-11001 |
| Alexa Fluor 594 rabbit secondary antibody | Invitrogen | Catalog no. A-11012 |
| DAPI | Vector Laboratories | Catalog no. H-1200 |
|  |  |  |
| Chemicals and recombinant proteins |  |  |
| Mycoplasma removal agent | MP Biomedicals | Catalog no. 930500044 |
| Epidermal Growth Factor (EGF) | Gibco | Catalog no. PHG0311L |
| Gentamicin | Gibco | Catalog no. 15750060 |
| Horse serum | Gibco | Catalog no. 26050088 |
| Fetal Bovine Serum (FBS) | Gibco | Catalog no. 12483020 |
| Antibiotic-Antimycotic (100X) | Gibco | Catalog no. 15240062 |
| Trypsin-EDTA (0.25%), phenol red | Gibco | Catalog no. 25200056 |
| Matrigel (Growth Factor Reduced) | Corning | Catalog no. 356231 |
| QIAGEN QIAamp DNA Mini Kit | Hilden | Catalog no. 56304 |
| Zylene | Ducsan | Catalog no. H29118 |
| Hydrogen peroxidase | FUJIFILM | Catalog no. 081-04215 |
| Gemcitabine | Lilly | Catalog no. 7502 |
| Nab-paclitaxel (Abraxane) | Celgene | Catalog no. 6200951B |
| Irinotecan | BORYUNG | Catalog no. 13120002 |
| Oxaliplatin | BORYUNG | Catalog no. K00200 |
| CellTiter-Glo 2D Cell Viability Assay | Promega | Catalog no. G9242 |
| CellTiter-Glo 3D Cell Viability Assay | Promega | Catalog no. G9638 |
| Ethanol | Merk Millopore | Catalog no. 1.00983.2511 |
| Methanol | Merk Millopore | Catalog no. 1.06009.2511 |
| Human Tumor Dissociation Kit | Miltenyi Biotec | Catalog no. 130-095-929 |
| Ham’s F-12 nutrient mix | Hyclone | Catalog no. SH30026.01 |
| Dulbecco’s Modified Eagle’s Medium | Hyclone | Catalog no. SH30243.01 |
| 4% Paraformaldehyde (PFA) | Biosesang | Catalog no. PC2031-100-00 |
| 10X PBS | Biosesang | Catalog no. PR4007-100-00 |
| Hydrocortisone | Sigma-Aldrich | Catalog no. H4001 |
| Insulin | Sigma-Aldrich | Catalog no. I9278 |
| Cholera toxin | Sigma-Aldrich | Catalog no. C8052 |
| Adenine | Sigma-Aldrich | Catalog no. A8626 |
| Amphotericin B | Sigma-Aldrich | Catalog no. A2942 |
| Rho-associated kinase (ROCK) inhibitor (Y-27632) | Sigma-Aldrich | Catalog no. Y0503 |
| LookOut Mycoplasma PCR Detection Kit | Sigma-Aldrich | Catalog no. MP0035 |
| Ultra-low gelling temperature (ULGT) agarose | Sigma-Aldrich | Catalog no. A2576 |
| Agarose | Sigma-Aldrich | Catalog no. A9918 |
| Citrate tribasic dihydrate | Sigma-Aldrich | Catalog no. S4641 |
| 5-Fluorouracil | Sigma-Aldrich | Catalog no. F6627 |
|  |  |  |
| Cell culture |  |  |
| 75T cell culture flask | SPL | Catalog no. 70075 |
| 96-well white plate | SPL | Catalog no. 30396 |
| 48-well cell culture plate | SPL | Catalog no. 30048 |
| 8-well cell culture slide | SPL | Catalog no. 30408 |
| 6-well cell culture plate | SPL | Catalog no. 30006 |
| 50ml conical tube | SPL | Catalog no. 50050 |
| 15ml conical tube | SPL | Catalog no. 50015 |
| 1.7ml microtube | Axygen | Catalog no. MCT-175-C |
|  |  |  |
| Oligonucleotides |  |  |
| KRAS forward primer | Bioneer | Catalog no. N/A |
| KRAS reverse primer | Bioneer | Catalog no. N/A |
| SMAD4 forward primer-1 | Invitrogen | Catalog no. hs00455111_CE |
| SMAD4 reverse primer-1 | Invitrogen | Catalog no. hs00455111_CE |
| SMAD4 forward primer-2 | Invitrogen | Catalog no. hs00532913_CE |
| SMAD4 reverse primer-2 | Invitrogen | Catalog no. hs00532913_CE |
| SMAD4 forward primer-3 | Invitrogen | Catalog no. hs00661591_CE |
| SMAD4 reverse primer-3 | Invitrogen | Catalog no. hs00661591_CE |
| SMAD4 forward primer-4 | Invitrogen | Catalog no. hs00765983_CE |
| SMAD4 reverse primer-4 | Invitrogen | Catalog no. hs00765983_CE |
| TP53 forward primer-1 | Invitrogen | Catalog no. N/A |
| TP53 reverse primer-1 | Invitrogen | Catalog no. N/A |
| TP53 forward primer-2 | Invitrogen | Catalog no. N/A |
| TP53 reverse primer-2 | Invitrogen | Catalog no. N/A |
| TP53 forward primer-3 | Invitrogen | Catalog no. N/A |
| TP53 reverse primer-3 | Invitrogen | Catalog no. N/A |
| TP53 forward primer-4 | Invitrogen | Catalog no. N/A |
| TP53 reverse primer-4 | Invitrogen | Catalog no. N/A |
|  |  |  |
| CRC organoids (YPAC cell lines) |  |  |
| YCLO-1 (YPAC-2) | Severance Hospital, Seoul, Korea | N/A |
| YCLO-2 (YPAC-5) | Severance Hospital, Seoul, Korea | N/A |
| YCLO-3 (YPAC-16) | Severance Hospital, Seoul, Korea | N/A |
| YCLO-4 (YPAC-17) | Severance Hospital, Seoul, Korea | N/A |
| YCLO-5 (YPAC-21) | Severance Hospital, Seoul, Korea | N/A |
| YCLO-6 (YPAC-23) | Severance Hospital, Seoul, Korea | N/A |
| YCLO-7 (YPAC-26) | Severance Hospital, Seoul, Korea | N/A |
| YCLO-8 (YPAC-27) | Severance Hospital, Seoul, Korea | N/A |
| YCLO-9 (YPAC-28) | Severance Hospital, Seoul, Korea | N/A |
| YCLO-10 (YPAC-29) | Severance Hospital, Seoul, Korea | N/A |
| YCLO-11 (YPAC-30) | Severance Hospital, Seoul, Korea | N/A |
| YCLO-12 (YPAC-31) | Severance Hospital, Seoul, Korea | N/A |
| YCLO-13 (YPAC-32) | Severance Hospital, Seoul, Korea | N/A |
| YCLO-14 (YPAC-34) | Severance Hospital, Seoul, Korea | N/A |
| YCLO-15 (YPAC-35) | Severance Hospital, Seoul, Korea | N/A |
| YCLO-16 (YPAC-36) | Severance Hospital, Seoul, Korea | N/A |
| YCLO-17 (YPAC-37) | Severance Hospital, Seoul, Korea | N/A |
| YCLO-18 (YPAC-39) | Severance Hospital, Seoul, Korea | N/A |
| YCLO-19 (YPAC-43) | Severance Hospital, Seoul, Korea | N/A |
| YCLO-20 (YPAC-44) | Severance Hospital, Seoul, Korea | N/A |
| YCLO-21 (YPAC-46) | Severance Hospital, Seoul, Korea | N/A |
| YCLO-22 (YPAC-47) | Severance Hospital, Seoul, Korea | N/A |
| YCLO-23 (YPAC-50) | Severance Hospital, Seoul, Korea | N/A |
| YCLO-24 (YPAC-52) | Severance Hospital, Seoul, Korea | N/A |
| YCLO-25 (YPAC-57) | Severance Hospital, Seoul, Korea | N/A |
| YCLO-26 (YPAC-58) | Severance Hospital, Seoul, Korea | N/A |
| YCLO-27 (YPAC-59) | Severance Hospital, Seoul, Korea | N/A |
| YCLO-28 (YPAC-60) | Severance Hospital, Seoul, Korea | N/A |
| YCLO-29 (YPAC-61) | Severance Hospital, Seoul, Korea | N/A |
| YCLO-30 (YPAC-65) | Severance Hospital, Seoul, Korea | N/A |
| YCLO-31 (YPAC-66) | Severance Hospital, Seoul, Korea | N/A |
| YCLO-32 (YPAC-67) | Severance Hospital, Seoul, Korea | N/A |
| YCLO-33 (YPAC-68) | Severance Hospital, Seoul, Korea | N/A |
| YCLO-34 (YPAC-69) | Severance Hospital, Seoul, Korea | N/A |
| YCLO-35 (YPAC-72) | Severance Hospital, Seoul, Korea | N/A |
| YCLO-36 (YPAC-75) | Severance Hospital, Seoul, Korea | N/A |
| YCLO-37 (YPAC-76) | Severance Hospital, Seoul, Korea | N/A |
| YCLO-38 (YPAC-77) | Severance Hospital, Seoul, Korea | N/A |
| YCLO-39 (YPAC-78) | Severance Hospital, Seoul, Korea | N/A |
| YCLO-40 (YPAC-79) | Severance Hospital, Seoul, Korea | N/A |
| YCLO-41 (YPAC-80) | Severance Hospital, Seoul, Korea | N/A |
| YCLO-42 (YPAC-82) | Severance Hospital, Seoul, Korea | N/A |
| YCLO-43 (YPAC-83) | Severance Hospital, Seoul, Korea | N/A |
| YCLO-44 (YPAC-85) | Severance Hospital, Seoul, Korea | N/A |
| YCLO-45 (YPAC-86) | Severance Hospital, Seoul, Korea | N/A |
| YCLO-46 (YPAC-87) | Severance Hospital, Seoul, Korea | N/A |
| YCLO-47 (YPAC-88) | Severance Hospital, Seoul, Korea | N/A |
| YCLO-48 (YPAC-89) | Severance Hospital, Seoul, Korea | N/A |
| YCLO-49 (YPAC-91) | Severance Hospital, Seoul, Korea | N/A |
| YCLO-50 (YPAC-92) | Severance Hospital, Seoul, Korea | N/A |
| YCLO-51 (YPAC-93) | Severance Hospital, Seoul, Korea | N/A |
| YCLO-52 (YPAC-94) | Severance Hospital, Seoul, Korea | N/A |
| YCLO-53 (YPAC-95) | Severance Hospital, Seoul, Korea | N/A |
| YCLO-54 (YPAC-96) | Severance Hospital, Seoul, Korea | N/A |
| YCLO-55 (YPAC-97) | Severance Hospital, Seoul, Korea | N/A |
| YCLO-56 (YPAC-98) | Severance Hospital, Seoul, Korea | N/A |
| YCLO-57 (YPAC-99) | Severance Hospital, Seoul, Korea | N/A |
| YCLO-58 (YPAC-100) | Severance Hospital, Seoul, Korea | N/A |
| YCLO-59 (YPAC-101) | Severance Hospital, Seoul, Korea | N/A |
| YCLO-60 (YPAC-102) | Severance Hospital, Seoul, Korea | N/A |
| YCLO-61 (YPAC-103) | Severance Hospital, Seoul, Korea | N/A |
| YCLO-62 (YPAC-104) | Severance Hospital, Seoul, Korea | N/A |
| YCLO-63 (YPAC-105) | Severance Hospital, Seoul, Korea | N/A |
| YCLO-64 (YPAC-106) | Severance Hospital, Seoul, Korea | N/A |
| YCLO-65 (YPAC-107) | Severance Hospital, Seoul, Korea | N/A |
| YCLO-66 (YPAC-108) | Severance Hospital, Seoul, Korea | N/A |
|  |  |  |
