# Supplementary material for: Establishing 3D organoid models from patient-derived conditionally reprogrammed cells to bridge preclinical and clinical insights in pancreatic cancer
Source: Mol Cancer. 2025 Jun 3;24:162. doi: 10.1186/s12943-025-02374-y (PMC12131615; doi:10.1186/s12943-025-02374-y)
Supplement: Supplementary file 5 — Supplementary Material 5 [file 12943_2025_2374_MOESM5_ESM.docx]

**Supplementary Table S4. Mutation profiles of KRAS, SMAD4 and TP53 in tissue, 2D and organoid culture conditions.**

| **No** | **Samples** | **Types** | **KRAS** | | | **SMAD4** | | | **TP53** | | |
| --- | --- | --- | --- | --- | --- | --- | --- | --- | --- | --- | --- |
|  |  |  | **gDNA** | **Protein** | **Mutation Types** | **gDNA** | **Protein** | **Mutation Types** | **gDNA** | **Protein** | **Mutation Types** |
| 1 | YCLO-32 | Tissue | c.35G→T | p.G12V | Missense | c.692delG | p.G231Afs*10 | Frameshift | c.585-588del  CCGA | p.I195MFx*51 | Frameshift |
|  |  | 2D | c.35G→T | p.G12V | Missense | c.692delG | p.G231Afs*10 | Frameshift | c.585-588del  CCGA | p.I195MFx*51 | Frameshift |
|  |  | Organoid | c.35G→T | p.G12V | Missense | c.692delG | p.G231Afs*10 | Frameshift | c.585-588del  CCGA | p.I195MFx*51 | Frameshift |
| 2 | YCLO-33 | Tissue | c.35G→A | p.G12D | Missense | Wild | Wild | Wild | c.817C→T | p.R273C | Missense |
|  |  | 2D | c.35G→A | p.G12D | Missense | Wild | Wild | Wild | c.817C→T | p.R273C | Missense |
|  |  | Organoid | c.35G→A | p.G12D | Missense | Wild | Wild | Wild | c.817C→T | p.R273C | Missense |
| 3 | YCLO-36 | Tissue | c.34G→C | p.G12R | Missense | c.403C→T | p.R135* | Nonsense | c.1024C→T | p.R342* | Nonsense |
|  |  | 2D | c.34G→C | p.G12R | Missense | c.403C→T | p.R135* | Nonsense | c.1024C→T | p.R342* | Nonsense |
|  |  | Organoid | c.34G→C | p.G12R | Missense | c.403C→T | p.R135* | Nonsense | c.1024C→T | p.R342* | Nonsense |
| 4 | YCLO-38 | Tissue | c.35G→T | p.G12V | Missense | c.227-231del  GAACA | p.R76lfs*26 | Frameshift | Wild | Wild | Wild |
|  |  | 2D | c.35G→T | p.G12V | Missense | c.227-231del  GAACA | p.R76lfs*26 | Frameshift | Wild | Wild | Wild |
|  |  | Organoid | c.35G→T | p.G12V | Missense | c.227-231del  GAACA | p.R76lfs*26 | Frameshift | Wild | Wild | Wild |
| 5 | YCLO-47 | Tissue | c.35G→A | p.G12D | Missense | Wild | Wild | Wild | c.742C→T | p.R248W | Missense |
|  |  | 2D | c.35G→A | p.G12D | Missense | Wild | Wild | Wild | c.742C→T | p.R248W | Missense |
|  |  | Organoid | c.35G→A | p.G12D | Missense | Wild | Wild | Wild | c.742C→T | p.R248W | Missense |
| 6 | YCLO-53 | Tissue | c.35G→T | p.G12V | Missense | Wild | Wild | Wild | c.742C→T | p.R248W | Missense |
|  |  | 2D | c.35G→T | p.G12V | Missense | Wild | Wild | Wild | c.742C→T | p.R248W | Missense |
|  |  | Organoid | c.35G→T | p.G12V | Missense | Wild | Wild | Wild | c.742C→T | p.R248W | Missense |
| 7 | YCLO-54 | Tissue | c.183A→T | p.Q61H | Missense | c.112dupA | p.R38Kfs*4 | Frameshift | c.642-643del  TA | p.H214Qfs*7 | Frameshift |
|  |  | 2D | c.183A→T | p.Q61H | Missense | c.112dupA | p.R38Kfs*4 | Frameshift | c.642-643del  TA | p.H214Qfs*7 | Frameshift |
|  |  | Organoid | c.183A→T | p.Q61H | Missense | c.112dupA | p.R38Kfs*4 | Frameshift | c.642-643del  TA | p.H214Qfs*7 | Frameshift |
|  |  |  |  |  |  |  |  |  |  |  |  |
| 8 | YCLO-57 | Tissue | c.183A→C | p.Q61H | Missense | Wild | Wild | Wild | c.916C→T | p.R306* | Nonsense |
|  |  | 2D | c.183A→C | p.Q61H | Missense | Wild | Wild | Wild | c.916C→T | p.R306* | Nonsense |
|  |  | Organoid | c.183A→C | p.Q61H | Missense | Wild | Wild | Wild | c.916C→T | p.R306* | Nonsense |
| 9 | YCLO-59 | Tissue | c.35G→A | p.G12D | Missense | c.334dupG | p.V112Gfs*2 | Frameshift | Wild | Wild | Wild |
|  |  | 2D | c.35G→A | p.G12D | Missense | c.334dupG | p.V112Gfs*2 | Frameshift | Wild | Wild | Wild |
|  |  | Organoid | c.35G→A | p.G12D | Missense | c.334dupG | p.V112Gfs*2 | Frameshift | Wild | Wild | Wild |
| 10 | YCLO-60 | Tissue | c.35G→T | p.G12V | Missense | Wild | Wild | Wild | c.801-802insT | p.N268* | Frameshift |
|  |  | 2D | c.35G→T | p.G12V | Missense | Wild | Wild | Wild | c.801-802insT | p.N268* | Frameshift |
|  |  | Organoid | c.35G→T | p.G12V | Missense | Wild | Wild | Wild | c.801-802insT | p.N268* | Frameshift |
| 11 | YCLO-61 | Tissue | c.35G→A | p.G12D | Missense | Wild | Wild | Wild | c.706T→A | p.Y236N | Missense |
|  |  | 2D | c.35G→A | p.G12D | Missense | Wild | Wild | Wild | c.706T→A | p.Y236N | Missense |
|  |  | Organoid | c.35G→A | p.G12D | Missense | Wild | Wild | Wild | c.706T→A | p.Y236N | Missense |
| 12 | YCLO-62 | Tissue | c.35G→A | p.G12D | Missense | Wild | Wild | Wild | c.723delC | p.C242Afs*5 | Frameshift |
|  |  | 2D | c.35G→A | p.G12D | Missense | Wild | Wild | Wild | c.723delC | p.C242Afs*5 | Frameshift |
|  |  | Organoid | c.35G→A | p.G12D | Missense | Wild | Wild | Wild | c.723delC | p.C242Afs*5 | Frameshift |
| 13 | YCLO-63 | Tissue | c.34G→C | p.G12R | Missense | Wild | Wild | Wild | c.747G→T | p.R2495 | Missense |
|  |  | 2D | c.34G→C | p.G12R | Missense | Wild | Wild | Wild | c.747G→T | p.R2495 | Missense |
|  |  | Organoid | c.34G→C | p.G12R | Missense | Wild | Wild | Wild | c.747G→T | p.R2495 | Missense |
| 14 | YCLO-65 | Tissue | c.35G→T | p.G12V | Missense | Wild | Wild | Wild | Wild | Wild | Wild |
|  |  | 2D | c.35G→T | p.G12V | Missense | Wild | Wild | Wild | Wild | Wild | Wild |
|  |  | Organoid | c.35G→T | p.G12V | Missense | Wild | Wild | Wild | Wild | Wild | Wild |
| 15 | YCLO-66 | Tissue | c.183A→C | p.Q61H | Missense | Wild | Wild | Wild | c.638G→T | p.R213L | Missense |
|  |  | 2D | c.183A→C | p.Q61H | Missense | Wild | Wild | Wild | c.638G→T | p.R213L | Missense |
|  |  | Organoid | c.183A→C | p.Q61H | Missense | Wild | Wild | Wild | c.638G→T | p.R213L | Missense |
